# Metal–organic framework-based materials for wearable energy harvesting and high-density energy storage technologies

**DOI:** 10.1039/d6ra05531a

**Published:** 2026-07-07

**Authors:** Tholkappiyan Ramachandran, S. V. Prabhakar Vattikuti, Yedluri Anil Kumar, Sunkara Srinivasa Rao, Laxman Singh, Kwun Nam Hui, Duc Anh Dinh

**Affiliations:** a Department of Physics, College of Science, United Arab Emirates University P. O. Box 15551 Al Ain Abu Dhabi United Arab Emirates thols2006@gmail.com; b School of Mechanical Engineering, College of Engineering, Yeungnam University Gyeongsan 38541 Republic of Korea vsvprabu@gmail.com; c Department of Condensed Matter Physics, Saveetha School of Engineering, Saveetha Institute of Medical and Technical Sciences, SIMATS Chennai 602105 India; d Department of Electronics and Communication Engineering, Koneru Lakshmaiah Education Foundation Bowrampet Hyderabad Telangana 500 043 India; e Department of Chemistry, Siddhartha University Kapilvastu Siddharth Nagar 272202 India; f Institute of Applied Physics and Materials Engineering, University of Macau, Avenida da Universidade Taipa Macau SAR China; g Center for Hi-Tech Development, Nguyen Tat Thanh University Saigon Hi-Tech Park Ho Chi Minh City Vietnam ddanh@ntt.edu.vn; h NTT Hi-Tech Institute, Nguyen Tat Thanh University Ho Chi Minh City Vietnam

## Abstract

Metal–organic frameworks (MOFs) constitute an exceptional class of porous crystalline materials, distinguished by their structural tunability, extensive surface areas, and multifunctional chemical attributes. Their intrinsic diversity and adaptability have positioned them at the forefront of numerous technological domains, including catalysis, gas sorption, and electrochemical energy storage. These applications are particularly significant in the context of wearable technologies, where mechanical flexibility, low weight, and high energy density are imperative. The inherent limitations of conventional energy-storage materials whether in geometric rigidity, insufficient energy density, or inadequate mechanical resilience underscore the pressing need to develop MOF architectures. This review synthesizes recent advancements in supercapacitors and battery systems, with a focus on the role of MOFs in enhancing device performance across key metrics such as energy density, charge–discharge kinetics, and operational long life. Owing to their thin-film compatibility and deformable profiles, these devices are poised for seamless integration into next-generation wearable platforms. MOFs, characterized by their dual functionality, mechanical compliance, and highly porous frameworks, exhibit significant promise in driving the evolution of wearable energy-storage technologies. Future progress will hinge on overcoming persistent challenges related to stability, scalability, and long-term performance. Continued advances in synthesis strategies, processing techniques, and fundamental understanding will be critical to unlocking the broader industrial potential of MOFs, spanning energy systems, pharmaceutical delivery, electronic devices, and environmental remediation. Ultimately, the incorporation of MOFs into wearable energy-storage systems may catalyze transformative developments in portable electronics, redefining both operational capabilities and user experience.

## Abstract 2

Metal–organic frameworks (MOFs) are porous crystalline materials with tunable structures, high surface areas, and versatile chemical properties.

## Graphical abstract

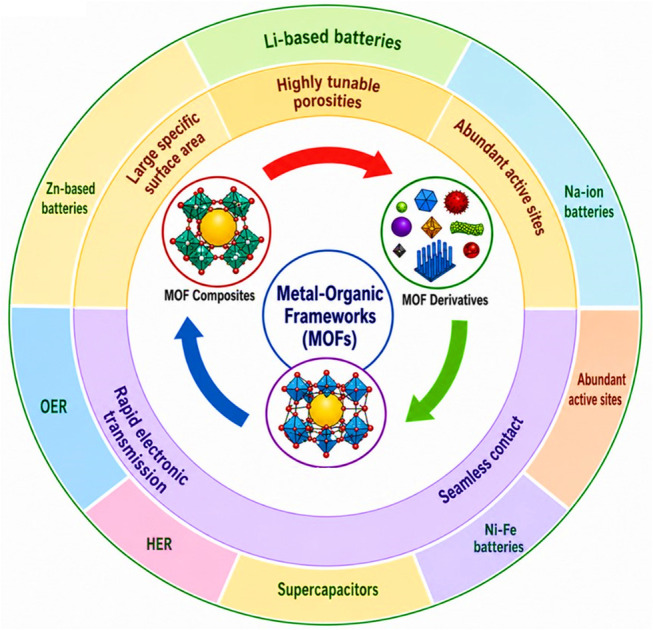

## 1. Introduction

**Fig. 1:**
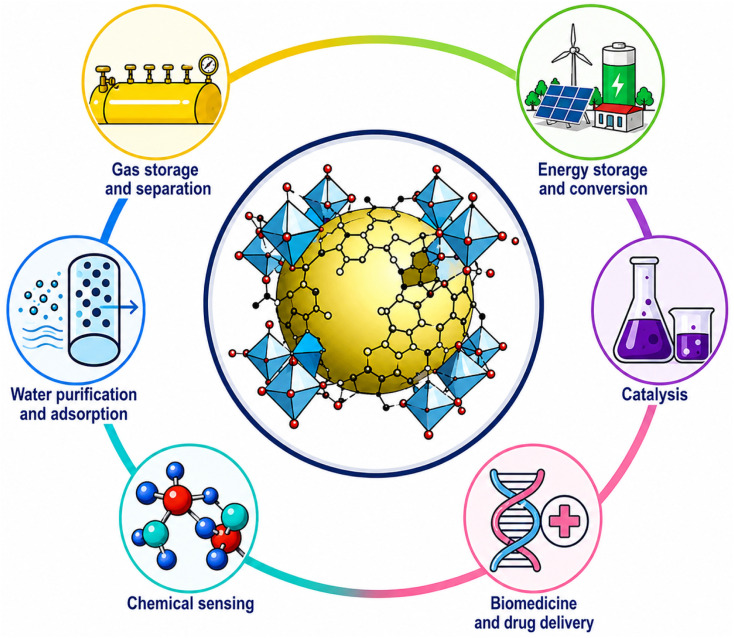
Applications of MOFs.

Metal–organic frameworks (MOFs) are crystalline materials best known for their immense porosity, readily harmonious internal construction and remarkable elasticity. These compounds comprise clusters and metal ions that are linked to structured connecting units in a coordinated network, giving MOFs unparalleled design versatility. Their chemical and physical qualities can be finely calibrated through judicious selection of the metal and organic constituents. Such precise configurability makes these solids highly suitable for diverse roles, especially those related to storing as well as releasing energy. The porous nature of MOFs and elastic framework combine to accommodate large volumes of gases or liquids within their porous engineered interiors for sodium ion batteries (SIB), potassium ion batteries (KIB), zinc ion batteries (ZIB), metal air batteries (MAB), lithium-ion batteries (LIB) and SCs (SCs). The applications of MOFs are depicted in Fig. 1. MOFs are porous crystalline materials combining metal ions and functional ligands and have a flexible structure and high surface area.^1^ Their application areas include catalysis, energy conversion, gas storage, drug delivery, sensors and energy storage.^2^ MOFs provide the raw material for active elements produced in powder form designed specifically for use as electrodes in electrochemical applications. However, the fabrication process necessitates the inclusion of polymer binders and additives to make the actual electrodes. Besides the MOFs can undergo structure variation according to temperature which could produce a diverse variety of nanoarrays sourced from MOFs. These materials are extremely interesting and also hold significant potential to use energy transition and storage.

The MOF-based nanoarrays produced from MOFs have gained widespread application in energy storage. Flexible electrical energy storage devices have gained significant attention in this period due to the increase in movable electronics, electric grids, medical devices, electric vehicles and the internet. These devices primarily power wearable devices and have the potential to revolutionize personalized electronics, healthcare and worldwide supply chains. There is a surge in research and development of wearable technologies, which have been widely adopted in takeout and flexible electronics, including curved smartphones, roll-up displays and various other movable as well as wearable electronic devices.^3^ A wide range of advanced energy storage devices are developed with exceptional versatility similar to lithium. MOFs have multi-functionality including combination of high specific surface area, porosity, structural compositionality, flexibility, functional versatility and the ability to control pore size and distribution. MOFs are currently regarded as very promising porous as well as crystalline organic–inorganic materials. The large number of synthesized MOFs are primarily offered in the form of powders. Notable concerns frequently encountered include the generation of abrasion dust, insufficient packing density and clogging or agglomeration problems. These disadvantages highlight the fact that incorporation in industrial productions is non-ideal. During recent years, researchers have attempted to convert these MOF powders into various forms such as beads, membranes, monoliths, gel/sponges and nanofibers.

One promising approach is the fabrication technique called three-dimensional (3D) printing which opens the possibility of the creation of complicated macroscopic structures from computer models.^4^ The primary emphasis is on the environmental uses, namely in the fields of water and gas treatment, encompassing filtration, adsorption and separation. Morphology control for 3D-printed MOF monoliths is done by direct-writing, seed-mediated *in situ* growth and coordination replication from a solid scaffold. They include the precursor, matrix incorporation, Selective Laser Sintering (SLS) and Digital Light Processing (DLP). MOF formation strategies gain a better understanding of the structural, chemical and textural properties of the 3D-printed MOF monoliths. SCs are important energy storage devices that can rapidly supply energy. In wearable electronic devices, SCs are mainly two types of flexible SCs (FSCs) and flexible electrodes (FEs). Presently, the FEs/FSCs come in various physical shapes and possess a wide range of functional features, resulting in a complex and vast system.^5^ Traditional healthcare services are centralized with clinical diagnoses and time-consuming blood tests using sophisticated and advanced equipment. This can affect patients who need urgent treatment and emergency treatment.

Emerging smart wearable sensors provide the ideal basis for improving health performance monitoring.^6^ These systems involve the use of skin or textile materials, making it easier to collect and analyze body fluids like sweat or saliva. They allow for prompt diagnosis and accurate treatment in a tailored way. The wearable sensors also remove the need for complicated pre-treatment procedures, sample preparation and constantly monitor the changing levels of important biomarkers. Wearable devices that are intended to be attached to the skin need to have specific physical characteristics, such as a low elastic modulus and the ability to withstand significant mechanical deformations of up to approximately 30%. The wearable devices should establish a strong connection with the skin to reduce the potential strain that may occur at the interface. In healthcare, the study is centred around creating a biocompatible, pliable platform that adheres to the skin and can measure the amounts of metabolites such as glucose, enzymes, amino acids, glucose, enzymes, amino acids and electrolytes. To integrate these devices into curved and 3D surfaces, it is necessary to use stretchable and flexible materials as substrates that have similar mechanical properties. Efficiently combining these adaptable sensors and energy storage devices is crucial for achieving optimal performance in response to the significant stress generated during normal bodily motion and muscle activity.^7^

## 2. Properties of MOFs

The goals of properties tuning are efficiency, sustainability and reliability on energy storage as the demand for sustainable resources increased.^8^ Many methods have been discovered and pursued by researchers to modify the structure and function of MOFs. Microwave-assisted technology can be used in the mechano-chemical processes required to produce MOFs with unique properties. The ability to change the metal and organic characteristics of MOFs is important. It enables the production of materials with good pore capacity, and this allows physical and chemical modifications.^9^ MOFs have many tunable properties, as shown in Table 1.

**Table 1:** Properties of MOFs

Property	Applications	Advantages	Limitations	Future scope
Structural flexibility	Wearable energy devices, flexible batteries	Adaptable to various shapes and movements	Potential loss of mechanical integrity over time	Development of more robust and durable flexible MOFs
Porosity	SCs, batteries, gas storage	High energy storage capacity	Possible collapse under stress or extreme conditions	Engineering MOFs with tunable porosity for enhanced performance
Electrical conductivity	Conductive fabrics, wearable electronics	Efficient energy transfer, faster charging/discharging	Usually low in pure MOFs	Hybridization with conductive polymers or materials
Ion transport	Batteries, SCs	Fast ion mobility, improved charge/discharge rates	Diffusion limitations in complex structures	Design of MOFs with optimized channels for ion transport
Thermal stability	High-temperature environments, outdoor wearables	Stability under varied temperature ranges	Degradation at very high temperatures	Synthesis of MOFs with enhanced thermal stability for extreme conditions
Mechanical strength	Durable wearable devices, smart textiles	Increased durability and resistance to wear	Trade-off with flexibility	Combining mechanical strength with flexibility through composite materials
Chemical stability	Wearables in harsh environments, medical devices	Long-term stability in various environments	Vulnerability to certain chemical environments	Enhancing chemical resistance through surface modifications
Flexibility	Stretchable electronics, conformal energy storage	Comfort and adaptability for wearables	Reduced mechanical strength	Innovations in flexible MOF composites with enhanced mechanical properties
Light weight	Portable energy devices, lightweight wearables	Increased user comfort, reduced load	The trade-off with energy density	Development of ultra-lightweight MOFs with high energy storage capabilities
Biocompatibility	Medical wearables, skin-contact sensors	Safe for long-term human contact	Limited options for biocompatible MOFs	Exploration of new biocompatible materials and coatings for MOFs
Energy density	High-capacity batteries, SCs	Longer-lasting energy storage	Potentially lower than traditional materials	Research into increasing energy density while maintaining flexibility
Power density	High-performance wearables, quick-charge devices	Rapid energy delivery	The trade-off with energy density	Balancing power and energy density through advanced MOF designs
Cycle life	Rechargeable wearables, long-term energy storage devices	Longevity and reliability	Diminished performance over time	Increasing cycle life through material optimization and protective coatings
Environmental friendliness	Sustainable wearables, eco-friendly electronics	Reduced environmental impact	Limited availability of green synthesis methods	Developing greener synthesis methods and recyclable MOF materials
Water stability	Outdoor wearables, moisture-resistant electronics	Stability in humid environments	Sensitivity to prolonged water exposure	Creating water-resistant MOFs through functionalization or hybrid materials
Tunable pore size	Customized energy storage, specific ion/molecule adsorption	Tailored storage capacity and selectivity	Complexity in synthesis and tuning	Refining control over pore size and distribution for specific applications
Multi-functionality	Wearable energy and sensing devices, smart textiles	Integrated functionalities (*e.g.*, energy storage + sensing)	Potential trade-offs between functions	Designing MOFs for multifunctional applications with minimal performance trade-offs
High surface area	SCs, gas storage, and separation	Increased energy and storage capacity	Potential structural fragility	Further increasing surface area without compromising structural integrity
Processability	Thin-film batteries, flexible electronics	Ease of integration into wearable formats	Complexity in large-scale production	Streamlining production processes for scalable and efficient MOF fabrication
Metal node diversity	Versatile energy storage solutions, customizable properties	Tailored properties based on metal choice	Variation in availability and cost of metals	Exploration of alternative and abundant metals for cost-effective MOFs
Ligand versatility	Tuning of properties for specific applications, custom MOF design	Customizable physical and chemical properties	Complexity in synthesis and control	Expanding the library of ligands for more versatile and application-specific MOFs
Charge storage capacity	High-capacity energy storage devices, SCs	Enhanced energy storage potential	Limited by framework collapse under high charge conditions	Increasing charge storage capacity through structural optimization and hybridization
Responsiveness to external stimuli	Smart textiles, adaptive energy devices	Sensing and adapting to environmental changes	Potential instability or degradation	Developing MOFs with robust and predictable responses to various stimuli
Scalability	Commercial production of wearable energy storage devices	Potential for large-scale manufacturing	Challenges in maintaining quality and properties at scale	Advancing scalable production methods while ensuring consistency and performance
Cost-effectiveness	Affordable wearable energy devices	Reduced overall cost for consumer wearables	High cost of some raw materials	Reducing costs through material substitution, green synthesis, and recycling of MOF components

## 3. MOF-based materials for wearable energy systems

MOFs have been utilized for diverse purposes due to their distinctive integrative and structural features.^10^ Significant focus has turned to the synthesis of MOF micro/nanomaterials with changeable shapes, boosting their performance in applications. MOFs with personalized compositions and porous structures, make effective models in high-temperature operations. They are crucial in building nanostructures with controlled pore shapes and large surface areas. Moreover, MOFs ensure strong chemical stability and offer a wide variety of functions, researchers started focusing on designing MOFs clean energy applications by combining LIBs, SCs, hydrogen production and storage process, for performance improvement, energy efficiency and environmental concerns.^11^ This will provide more practical and cost-effective solutions for energy storage technologies. For optimal efficiency and performance, energy storage systems need higher charge/discharge rates, greater theoretical capacities and better electronic stability. MOF-based electrodes hold significant potential with several benefits by using MOFs adjustable porosities as effective active sites. In addition, MOFs have fast response time, means that it can quickly respond to various stimuli by the benefit of adjustable porosity, gives them the ability to change size.^12^ The MOFs can carry out electrical transport, display magnetism and store gas or hazardous molecules. Overall, they possess the potential for an array of applications. Table 2 depicts different MOFs and other conventional materials for energy storage.

**Table 2:** Metal–organic frameworks and other conventional materials for energy storage

Material class	Material type	Porosity	Electrical conductivity	Energy density	Charge/discharge rate	Surface area	Cyclability	Environmental impact	Representative references
Pristine MOFs	Metal–organic frameworks	Very high due to well-defined porous crystalline structures	Generally low (10^−10^–10^−6^ S cm^−1^ for most MOFs)	Moderate	Moderate to slow owing to poor intrinsic conductivity	Extremely high (typically 500–7000 m^2^ g^−1^)	Moderate; structural degradation may occur during long-term cycling	Synthesis may require organic solvents and energy-intensive processing	13 and 16–18
Conventional inorganic materials	Traditional metal oxides (MnO_2_, Co_3_O_4_, Fe_2_O_3_, NiO, *etc.*)	Low to moderate	Insulating to semiconducting	Moderate to high	Moderate	Moderate (10–300 m^2^ g^−1^)	Generally good	Depends on metal source and processing route	13 and 19
Conductive polymers	PANI, PPy, PEDOT and derivatives	Low to moderate	High intrinsic conductivity	Moderate	Fast	Low to moderate	Moderate; volume expansion can reduce long-term stability	Moderate environmental impact	14 and 20
Carbon-based materials	Graphene, CNTs, activated carbon, carbon nanofibers	Moderate to high depending on activation and structure	High	Moderate to high	Fast	High (500–3000 m^2^ g^−1^)	Excellent	Relatively low environmental impact	15 and 21
MOF composites	MOF/carbon, MOF/polymer, MOF/metal oxide composites	High	Moderate to high due to conductive secondary phase	High	Fast	High	Improved compared with pristine MOFs	Depends on composite constituents	22–25
MOF-derived materials	Carbonized MOFs, metal oxides/sulfides/phosphides derived from MOFs	Moderate to high	High	High	Fast	High	Excellent	Moderate	26–29
MOF-containing flexible devices	Flexible MOF-based electrodes, supercapacitors, and batteries	Depends on active material	Device-dependent	Device-dependent	Device-dependent	Device-dependent	Device-dependent	Depends on fabrication process	30–33

### 3.1. Durability of MOFs in energy storage and conversion

Flexible MOFs have a rare structure-changing ability, which distinguishes them from other solid-state materials.^16^ These unique properties make them effective in many fields such as molecular separation, optoelectronic devices, chemical sensing, data storage and biomedical applications. This is possible because crystalline materials have a constant height during the transition period. By integrating supporting groups into molecular arrays, planarization can be attained and the efficacy of flexible MOFs can be enhanced by meticulous design. Soft, porous crystals, often referred to as flexible MOFs, are crystalline materials that display dynamic behaviour in response to external chemical and physical stimuli. These stimuli might include light, heat, electric or magnetic fields, or any other material. Unlike other solid-state materials, flexible MOFs have the unique ability to modify their structure with remarkable flexibility for variety of applications, including molecular separation, optoelectronic devices, chemical sensing, data storage and biological applications. This is feasible because, in the transitional phase, crystalline materials can display significant degrees of order.^17^ Supporting groups can be incorporated into molecular arrays to enable the design to be realized and to carefully enhance the performance of flexible MOF. When exposed to external chemical and physical stimuli like heat, light, magnetic or electric forces or certain chemicals, flexible MOFs behave well while preserving their crystalline state.^18^

### 3.2. Flexibility of MOFs and its impact on wearable technologies

Although MOFs are usually considered to be rigid, crystalline structures, some of them are unique structures that show dynamic properties upon external stimuli, referred to as flexible MOFs or soft, porous crystals (SPCs). In contrast, rigid porous materials have fixed frameworks with fixed pore sizes and fixed crystal structure, while flexible MOFs can change the geometric structure of the framework, the size of the pores, and the crystal structure reversibly without changing the overall framework connection. Kitagawa and co-workers have extensively studied this behavior and it has become an important feature of MOF chemistry for applications such as adsorption, separation, sensing, catalysis, and energy storage. Flexibility in framework is due to the dynamic interactions occurring between the metal nodes and organic linkers, which enable the framework to undergo structural rearrangements under certain conditions. These structural changes can be induced by adsorption of guest molecules, changes in temperature, pressure, electric field, light irradiation, and chemical stimuli. This is the reason why the pore environment of flexible MOFs can change dynamically with external stimuli, which gives rise to some unusual and novel properties in MOFs with respect to their rigid porous materials. Their response to environmental changes has shown a tremendous interest in the potential of MOFs in advanced energy-storage and energy-conversion technologies.

Based on the flexibility mechanisms, flexible MOFs can be generally divided into three types. Breathing MOFs are a class of MOFs with large and reversible changes in the unit cell dimension and pore volume upon guest adsorption and desorption. The examples include the MIL-53 and MIL-88 families of which the pore size change from narrow-pore to large-pore and *vice versa* occurs without destruction of the crystalline framework. These materials may be promising candidates for electrochemical applications due to dynamic changes in pore structure that may aid in ion diffusion and electrolyte penetration. The second type are the gate-opening MOFs, having partially closed pore apertures that open when the guest molecule or external stimuli is present. ZIF-8 and ZIF-7 show gate-opening behavior due to linker motion and flexibility of the framework. Such phenomenon can lead to selective molecular transport and controlled accessibility of active sites that can enhance ion transport and charge storage properties of electrochemical systems. Important class of MOFs are ligand-flexible MOFs in which flexibility is mostly due to rotation or conformational changes of the organic linkers. In such systems there is little change in the overall topology, but there is a degree of framework adaptation. Adjustable pore environment can be achieved by ligand dynamic motion, thus allowing the guests to diffuse and improving host–guest interactions. Such structural adaptability can be beneficial for improving electrochemical kinetics and ion-storage processes.

Some MOFs are stimuli responsive, meaning that the structure changes in response to thermal, mechanical, electrical, optical or chemical stimuli, in addition to the breathing and gate-opening mechanisms. Such adaptive materials may change their porosity, conductivity or adsorption properties upon exposure to environmental changes, providing possibilities for the creation of intelligent and multifunctional energy-storage devices. While there is a concept of intrinsic flexibility in the framework of MOF chemistry it is essential to differentiate this from mechanical flexibility of wearable devices. Flexibility in the framework is determined by how the molecular and/or crystallographic structure changes, while flexibility in the device is determined by how the size of the electrodes, films, fibers, textiles, or integrated energy storage systems change when subjected to bending, twisting, stretching, folding, or compression at the macroscopic level. These are connected concepts but have some differences in them. Even if the MOF is rigid, the device can be mechanically flexible if the MOF is incorporated in a flexible material like a supercapacitor or battery, and conversely, an intrinsically flexible MOF may not necessarily create a mechanically flexible device. This is especially significant as many of the studies mentioned in the wearable-energy literature utilize MOF composites, MOF derived materials, or flexible electrodes containing MOFs as opposed to intrinsically flexible MOFs. For instance, UiO-66 is regarded as a structurally rigid MOF with good chemical stability and has been extensively applied to flexible energy-storage devices. Likewise, ZIF-67 is often used as a precursor material to synthesize porous carbons and transition-metal compounds, instead of using it as a traditional flexible MOF. Materials like MIL-53, MIL-88 and ZIF-8, however, present different levels of flexibility in their framework structure, and thus serve as valuable models to study the effects of dynamic structural behavior on electrochemical properties.

**Fig. 2:**
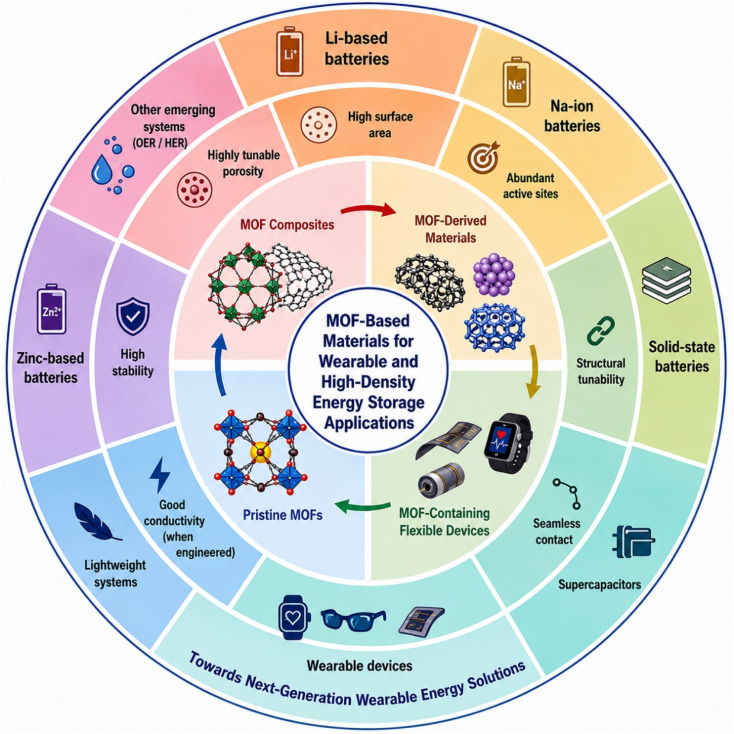
Overview of MOF-based materials for wearable energy-storage systems.

The importance of flexibility in the framework for energy-storage applications stems from a number of factors. Dynamic pores could allow ions to move by decreasing the diffusion path length and increase the accessibility to the electrolyte. Structural reversible adaption can also compensate for volume changes in charge–discharge process, which will reduce the mechanical stress during charge–discharge process and enhance the cycling stability. In addition, continuous activation of the active sites through breathing (or gate-opening) can improve the charge-storage efficiency and electrochemical utilization. These properties indicate the potential for enhanced electrochemical characteristics, especially for applications that demand high ion mobility and extended cycling life. However, most of the wearable energy-storage devices reported so far are based on the synergistic synergy of MOFs and conductive polymers, carbon nanomaterials, metal oxides or flexible substrates. Thus, this review does not only focus on the intrinsically flexible MOFs but also covers a wider variety of MOF-based materials such as pristine MOFs, MOF composites, MOF-derived materials, and MOF-containing flexible devices. This wider context gives a more complete picture of the potential of MOF chemistry for solving challenges for next generation high-density and wearable energy storage systems. Fig. 2. Relationship between MOF-based materials and wearable energy-storage applications.

## 4. MOFs for wearable energy harvesting

**Fig. 3:**
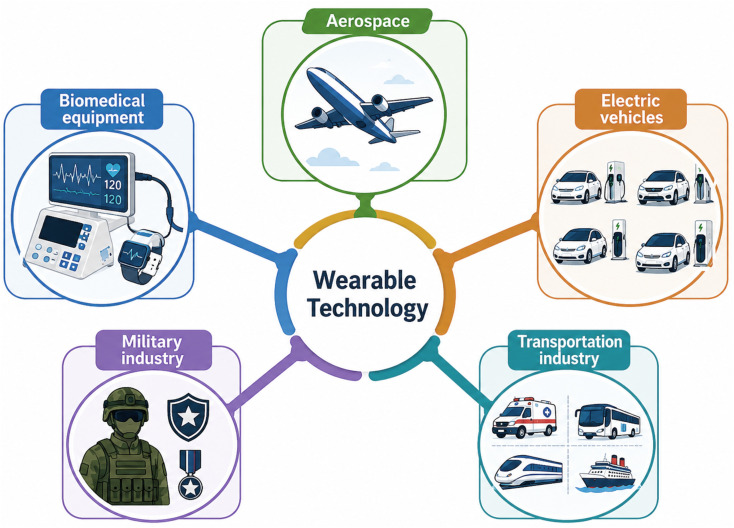
Wearable technologies and its related application fields.

Wearable technology is defined as devices that are worn on the body, usually having integrated sensors that connect to the internet to track health and fitness as well as use smart technology in everyday settings.^22^ Grid-connected energy harvesting and consumption on a large scale is not the sole applicable in the field of energy. Energy conversion systems are necessary for integration with mobile components of instrumentation, robotics, durable field machines, vehicles and durable field devices that can be categorized as energy sources which exhibit high degrees of external stress tolerance. Extremely adaptable devices provide seamless bonding with curved substrates. Scientists utilized various techniques to create stretchy electronic devices that convert stretching motions into nanoscale bending strain.^23^ The gadgets' flexibility can be enhanced by converting them into a stretchable form. By adding buckling and coiling properties, the bending strains in the flexible devices can be transformed into tensile strains. Most conductive metal thin films exhibit a degree of flexibility, often with radii of curvature in the range of a few millimeters, when applied to a flexible substrate. Fig. 3 shows wearable technologies and its related application fields. Percolated thin film creation is a method employed to combine conductivity and stretchability. CNT, graphene and coatings can be deliberately broken into pieces and arranged on flexible surfaces. During the process of straining, the conductive layers experience fractures that form plate-like structures, leading to the creation of interconnected networks. A straightforward approach incorporates the inflexible active substances into flexible compounds to fabricate devices that can be stretched. Typically, stretchy polymers are used to inject high volumes of nano-sized semiconducting metallic molecules, such as silver, or carbon materials like CNTs, or a hybrid of CNT-Ag. These inherently elastic devices can endure significant strain while maintaining their generating capabilities or energy storage without impacting their electric conduction.^24^

Breakthroughs were highlighted in the integration of MOFs with wearable technologies, particularly in energy conversion systems. Such breakthroughs are significant as wearable devices demand efficient, lightweight and flexible power sources, while MOFs offer distinguishing properties, such as high surface area and tunable porosity. The latest innovations built around MOFs emphasized the utilization of MOF's properties for enhancing energy conversion in photovoltaic, thermoelectric and piezoelectric systems, which are crucial for powering wearable devices. The breakthrough highlighted here also focuses on the application of MOFs in photovoltaic energy conversion: for the first time, research successfully doped MOFs into organic solar cells (OSCs), significantly increasing their efficiency and stability. The addition of MOF-derived carbon materials in electron transport layers is already evident to improve charge mobility in OSCs, with power conversion efficiencies above 18%. This development makes solar panels not only more efficient but also allows them to be put on or in clothing and wearable electronics without losing comfort, flexibility or weight. Advances in MOFs that are simultaneously flexible, electrically conductive and transparent have made transparent solar cells integrated into fabrics possible. MOFs are significantly changing the landscape in the growing field of thermoelectric energy (TENGs) conversion by tapping into body heat. Due to their flexibility, MOF-based TENGs can be stuck directly onto the skin to convert body heat into electricity.

More recently, power factors as high as 2.0 mW mK^−2^ for doped MOFs have been reported, which would permit uncharged wearable devices with years' extended duration of use from device batteries. A new area opened by combining MOFs with piezoelectric materials is that of energy harvesting. MOFs doped with piezoelectric nanogenerators can convert mechanical movement that includes walking and jogging into electrical energy in wearable devices. MOF structure optimization has already been shown to enhance piezoelectric output in MOFs compatible with self-powering wearables capturing exercise activity. Hybrid systems consisting of MOFs that are combined with a variety of energy conversion technologies have gained specific attention in research. A new device has been designed that integrates the MOF-based solar cell with the thermoelectric generator that will capture both solar energy and body heat. This approaches maximal energy capture and enhances the overall efficiency of the system so that wearables can power sensors and display without needing to be recharged all too often. Energy storage solutions for wearables have also been very popularly used and well employed using MOFs. Researchers use MOFs in SCs and batteries to develop more lightweight and flexible energy storage devices. MOF-based electrodes enjoy good capacitance and cycling stability, making them an excellent option for energy storage applications. This is why wearables can store energy received from different sources and provide a reliable power supply. MOFs can now be embedded into textiles, which means that a smart cloth with the potential to harvest and store energy with soft comfort can be produced. Scientists have successfully integrated MOF composites into fabric structures to create charges in devices. There has been considerable recent progress made towards integrating MOFs with wearable technologies for energy conversion. Self-sustaining wearables, hybrid energy systems and MOF-based energy storage solutions will find new applications in photovoltaic, thermoelectric, and piezoelectric technologies promising wearable technologies that might result in added convenience, sustainability and user experience.

### 4.1. Photovoltaic systems

Improvements in photovoltaic energy conversion for wearable systems have seen increased efficiencies and longevity and better application of solar technology in smart wearables over the past year up to 2024. On flexible substrates, efficiencies greater than 25% have been achieved with roll-to-roll processing of flexible perovskite solar cells to realize lightweight and conformable solar cells for integration into clothing. These researchers were also able to make significant advances into transparent conductive coatings incorporating the use of materials like graphene, which can produce energy without any dilution of the esthetic value. Improved encapsulation techniques have enhanced the stability and strength of photovoltaic devices to resist environmental effects such as moisture and mechanical stress. Hybrid energy harvesting has been boosting the efficiency of the entire system combining solar energy with thermoelectric generators. Thus, wearable devices can harvest energy from both the sun and body heat. Advances in power management have ensured that energy use is optimized for operation under variable conditions of light. Additionally, the integration of lightweight energy storage solutions enhances device reliability. This incorporation of Internet of Things (IoT) technologies in wearable photovoltaic systems allows for real-time monitoring and measurement of generated and consumed energy, further advance performance optimization. These workplace photovoltaic wearables as vanguards of sustainable energy, the inconsiderate endeavor of creating efficient, durable and versatile forms for next-generation technologies.

From an environmental energy point of view, solar energy is another form of energy that can be used in powering wearable energy systems. Generation photovoltaic energy can be integrated with several platforms for use in several application scenarios. In photovoltaic devices, incident radiation in the active material brings about the generation of electron–hole pairs which are then swept and collected at the electrode. Although there are numerous research on dye-sensitized solar cells, the characteristics of graphene allow it to become employed as a transparent, conducting electrode.^25^ They are distinguished from traditional silicon-based modules due to their relatively lower cost, ease of flexibility and reduced weight, making them ideal for self-powered wearable devices. However, the mechanical strength of the polymer solar cells has been compromised, and the efficiency of the solar cells has decreased. Polymer solar cells are sensitive to photochemical degradation thus; they are not stable. By employing organic photovoltaics and CNT/polymer-based SCs the design concept was expanded to an electrically active flexible and thin charging element. The total efficiency of this device is 6% with less than 50 mm thickness. The organic solar cell (OSCs) application of this device is flexible and stable it can be regarded as a portable and long-lasting power supply for wearable electronics.^26^

An OSC significantly refers to a photovoltaic cell made of organic electric materials. Organic and polymer electronics has always been one of the key research areas in which the group deals with semiconducting organic polymers or mini organic molecules. There are many inherent uses of organic electronics and the use in solar cells is quite popular. In this regard, they can address issues of redundancy and difficulties of stability in efforts aimed at revealing future solar batteries that are cheap and versatile. OSCs offer cheaper, thin as well as convenient solar modules than silicon thus possibilities of recharging wearable devices. However, mechanical properties and the power requirements of the sensors can often be met, and the type of solar energy can be determined using flexible organic solar cells or silicon solar cells depending on the application of the measurement system. It is suitable for efficient printing processes and can be used in the fiber method for textile substrates, making it suitable for use as an energy source. However, solar cells are still limited by lighting conditions. The developments in thinner copper indium gallium selenide (CIGS) technology have made it possible for the cells to continue producing energy in low light conditions because CIGS only works very well in good lighting. Photochemical stability is significantly more compromising. However, the technology connected with photoelectric energy conversion appears to be comparatively well-developed availing privileges inclusive of high-power density as well as a marvelous amount of output current.^27^

### 4.2. Thermoelectric systems

The conversion of thermal energy method harnesses the thermoelectric effect of generating higher electricity. The temperature difference between the body and its surroundings can be utilized in stationary situations. Researchers have recently discovered electric method for harvesting low-grade heat using thermosensitive crystallization-boosted liquid thermocells.^28^ This breakthrough could potentially allow for the harvesting of body heat energy at room temperature. However, it is important to note that a single thermoelectric junction can only generate a small amount of power. To avoid such a limitation, one has to connect several hundreds of thermoelectric P–N junctions in series to obtain a reasonable amount of power for operating the sensor. Hence the size of the device is quite large compared to other conventional devices that can perform similar functions. Flexible thermoelectric systems can be obtained from the use of flexible substrates, flexible connections through liquid metal, fibre-optic materials and smart textiles, besides flexible two-dimensional (2D) materials.^29^ Their application in wearable electronics is based on screen printing of organic materials (such as a thin coating of poly (3,4)) or microgenerators on flexible substrates can be used to create flexible electronic devices like TENGs.

Advances during the last few years in thermoelectric power generation for wearable devices have been quite promising, converting body heat into usable electrical energy. It becomes especially important as more complex wearable technologies require continuous power supply without frequent recharging. Research work has been focused on the optimization of flexible and lightweight thermoelectric materials to enhance efficiency and integration in wearable formats. Recent studies show that the development of new materials, such as polymer-based thermoelectric composites, has been known to raise *ZT* values as high as 1.4. This would provide higher efficiency for the process of heat into electricity, meaning wearables could run continuously only on human warmth. Conventional thermoelectric materials combined with 2D materials like graphene and transition metal dichalcogenides have thus far led to improvements in thermoelectric properties. This hybrid materials exhibit flexibility along with enhancement of thermal conductivity and electrical insulation, making them promising candidates for direct skin application. Recent studies have reported that flexible TENGs could be mounted to the skin to achieve a power output sufficient to charge wearable devices, such as a fitness tracker or smartwatch. These TENGs pick the temperature differential of skin and its environment; hence, they produce power without any external sources of energy. In addition, recent advancements in microfabrication have enabled the development of very miniaturized forms of thermoelectric devices that can be directly implanted into fabrics. Recently, body heat can be converted into electrical power; hence, it indicates the possibility of self-sustaining wearable electronics. Overall, the recent advancements regarding thermoelectric power generation for wearable devices should increase both utility and convenience in personal electronics, hence widely paving the way to an energy-efficient future.

### 4.3. Fuel cells

A fuel cell is an electronic device that converts the electrical energy of the oxidant and fuel into electrical energy. Types of fuel cells (FCs) include those by their electrolyte and the time taken to start up. Standard fuel cells are large and dense. Examples include phosphoric acid, alkaline and solid oxide fuel cells.^30^ On the other hand, several of the later generations of fuel cell technologies, for instance flexible PEMFCs, MFCs, EBCs and DMFCs have faster start-up times and can be used directly as portable system power sources. Electrochemical Biofuel Cells (EBFCs) are not constant, and biofuels have comparatively less energetic conversion efficiency due to less mechanical strength and chemical stability of the electrodes used. Thus, FC electrochemical energy conversion technology is widely recognized for having an opportunity to create a power source that is characterized by a high power-to-weight ratio. The device can be designed to be able to fold to an almost film-like electronic skin, a tattoo-like look. It may be coated on a textile base or incorporated in the fabric in the form of yarn fabric. This makes the structure highly reversible as well as flexible and with great tensile strength. However, as with all fuel cells, they have a finite working life and the fuel must be continuously supplied to the cell which makes it more suitable for use in other areas of biomedical implant.^31^

### 4.4. Mechanical energy harvesting

The procedure of changing mechanical energy into electrical power is known as mechanical energy harvesting. Thus, while discussing mechanical energy harvesting meso-level viewpoints are necessary. The conversion of mechanical motion into electrical motion has been broken down into three main categories, First is a technology known as electrostatic energy harvesting, another approach is piezoelectric energy harvesting, and the third category is electromagnetic energy harvesting.^32^ Energy from wind is another form of power generation that uses the wind to rotate wind turbine blades to turn an electric generator that converts the mechanical energy obtained from the wind into electrical energy. This is considered another form of electromagnetic power generation. Nanogenerators are special devices by which energy is transformed from mechanical energy into electrical energy. Operating at micro-and nanoscales, they employ piezoelectric and triboelectric methods. The pyroelectric generator distinguishes itself from nanogenerators by function to harness thermal energy from time-varying temperature fluctuations. It does not rely on relative temperature differences like a triboelectric nanogenerator (TENGs). The first category involves the use of electrostatic harvesters. For these devices, there is a dependence on pre-charged electrets and vibration-induced capacitance change. The second category involves the usage of TENGs.^33^

The status of mechanical energy harvesting for wearable devices has considerably advanced in recent times to convert human movement into usable electrical energy. This technology “harvests” energy from walking and running or even simple movements to idealize powering wearable devices without batteries. Piezoelectric materials advanced in the field have been integrated into wearables. These materials generate an electric charge when experiencing mechanical stress, thereby increasing the efficiency of energy conversion. Recent studies have recently revealed that even at low stresses, nanostructured piezoelectric materials like PZT and PVDF can generate impressive amounts of energy. Researchers have also demonstrated wearable piezoelectric generators by reporting energy densities beyond 5 mW cm^−2^ for powering sensors and small electronics embedded in clothes.^34^ TENGs have gained good interest as a mechanical energy harvesting complement technology. TENG makes use of the contact electrification phenomenon between two dissimilar materials to produce electricity. The state-of-the-art innovations include optimization of the materials and structures for the TENGs. This results in efficient energy harvesting devices tailored according to different types of motion, such as bending and stretching. Some of the recently developed TENG prototypes have achieved efficiencies of as high as 50% in energy conversion, which further increases the appeal of such devices for use in wearable electronics. There is also the incorporation of smart textiles with the capability of energy harvesting. Those textiles capture energy from the movement of the body and convert these into electrical power, thus opening doors for self-sustaining wearables. All mechanical advancements in energy harvesting pave the way for the development of innovative, energy-efficient wearable technologies, thus enhancing the user experience through reductions in reliance on traditional power sources.

### 4.5. Power generation

Wearable gadgets must possess flexibility and elasticity to accommodate the wearer's various body positions.^34^ Wearable inclinations designed for outdoor; sports and industrial environments must adhere to the waterproof and dustproof standards outlined in the ingress covering code. In addition, wearable gadgets essentially include intelligence, meaning they should be built on a sophisticated platform that can seamlessly integrate with the IoT framework. Therefore, the system must include a microcontroller, one or more power supplies, several sensors and a wireless transceiver. The first requirement for high-tech use is minimum energy consumption. Sensors and wireless transmitters are essential for energy consumption in any system architecture. Therefore, design challenges are important in the integration of various applications.^35^ The recent breakthroughs in power supply for wearable gadgets greatly improved their functionality and usability, especially in outdoor and sports environments as well as in industrial settings.

The wearable devices should meet the standards of the IP code, ensuring dustproof and waterproof capabilities, which has made them very durable and reliable under any kind of circumstances. Recently, innovations have been devoted to integrating sustainable sources of energy to provide support for the growing demand for wearable devices that seamlessly connect to the IoT.^36,37^ The first area is the improvement of energy harvesting technologies, from flexible photovoltaic (PV) cells to wearable solar cells. This solar cell is one of the easier-to-integrate photovoltaic devices in apparel and accessories, providing a lightweight and efficient source of power generation. Modern OSCs recently reached conversion efficiencies of over 10%. Enough energy can now be harvested from them to power wearable electronics while sustaining flexibility and comfort. Apart from these, other developments have been realized in the field of thermoelectric power generation that empowers wearable technology to capture and convert body heat into electrical energy. New materials have highly improved these systems in efficiency so they can use small temperature differences to create power, useful for wearables tracking health metrics or the amount of physical activity, which can keep generating power during use. Combining piezoelectric generators that can convert mechanical movements into electricity with photovoltaic and thermoelectric technologies will ensure higher output and higher efficiency in terms of energy provided. This multifaceted approach will ensure that there is always a power supply for wearables, thus maximizing performance while minimizing the charging needed to be drawn from an external source. Table 3 depicts the overview of different wearable technologies using MOFs.

**Table 3:** Overview of different wearable technologies using metal–organic frameworks

Application	MOF classification	Advantages	Limitations	Role of MOFs	Representative examples	Future prospects	Representative performance	Stability	Ref.
Photovoltaic energy conversion	MOF composites and MOF-containing flexible devices	Low cost, lightweight, flexible, textile integration	Photochemical degradation, reduced mechanical durability, dependence on illumination conditions	Interfacial engineering, charge transport enhancement, defect passivation, stability improvement	Organic solar cells (OSCs), DSSCs, perovskite solar cells, flexible CIGS cells, graphene-based solar cells	Hybrid MOF-photovoltaic systems with enhanced efficiency and long-term stability	Power conversion efficiency typically 10–20% depending on device architecture	>80% efficiency retention after ∼1000 bending/operation cycles	34
Thermoelectric power generation	MOF composites	Harvests body heat, wearable integration, lightweight design	Low conversion efficiency, large-area requirements for higher output	Enhancement of Seebeck coefficient, phonon scattering control, thermal conductivity reduction	Flexible TENGs, wearable thermoelectric devices, thermoelectric textiles, liquid thermocells, organic thermoelectric materials	Miniaturized self-powered wearable systems	Power density up to ∼10 µW cm^−2^ under wearable conditions	>90% performance retention after >1000 cycles	35
Fuel cell energy conversion	MOF-derived materials and MOF composites	High power-to-weight ratio, wearable compatibility, rapid startup	Fuel supply requirement, finite lifetime, catalyst degradation	Electrocatalysts, catalyst supports, proton-conducting frameworks	Flexible PEMFCs, microbial fuel cells, DMFCs, SOFCs, biofuel cells	Advanced MOF-derived electrocatalysts for sustainable energy systems	Peak power density >500 mW cm^−2^	Stable operation for >1000 h	36
Mechanical energy harvesting	MOF composites and MOF-containing devices	Multiple harvesting mechanisms, wearable adaptability	Variable efficiency, design complexity	Piezoelectric/triboelectric enhancement, mechanical reinforcement, charge trapping	Piezoelectric nanogenerators, electromagnetic harvesters, TENGs, electrostatic harvesters, wind-energy harvesters	Hybrid self-powered systems combining harvesting and storage	Output power up to 100 µW cm^−2^	>80% output retention after >1000 cycles	37
Power generation systems	MOF-containing flexible devices	Suitable for IoT, healthcare, sports, and outdoor electronics	Waterproofing, dustproofing, high power demands of electronics	Power management, energy conversion and storage integration	Fitness trackers, health-monitoring devices, smart glasses, AR/VR wearables, IoT-enabled outdoor devices	Fully integrated smart wearable energy platforms	System energy-management efficiency >90%	Stable operation over >500 cycles	38
Energy storage devices	Pristine MOFs, MOF composites, and MOF-derived materials	High surface area, tunable porosity, lightweight, multifunctional	Low conductivity of pristine MOFs, integration challenges, scalability concerns	Electrode materials, electrolyte modifiers, conductive frameworks, redox-active sites	Flexible supercapacitors, wearable Li-ion batteries, graphene-based storage devices, solid-state SCs, hybrid storage systems	Advanced MOF architectures for high-energy-density flexible electronics	Specific capacitance typically 100–1500 F g^−1^ depending on material type and testing conditions	>90% capacity retention after >5000 cycles	39

## 5. MOFs for wearable energy storage

In recent times, the electronic industry has advanced and there is a growing appeal for portable gadgets that offer both wearability and multi-functionality. Wearable energy storage devices are indispensable for portable electronic devices. Advanced SCs are currently used electronic devices to meet the increasing demand for future electronic devices.^36^ The demand for high-performance wearable energy storage devices, including lightweight, flexible, good electrochemical performance and compatibility with fabrics and materials, is driving the research in energy transfer systems. The implementation of the above tasks is particularly relevant to the electrode material, which is also the main function of SCs. Table 4 depicts the various energy storage devices for wearable systems.

**Table 4:** Energy storage devices for wearable systems

Device category	Technology	MOF classification	Key features	Mechanism	Representative performance	Wearable applications	Ref.
Energy storage	Supercapacitors (SCs)	MOF composites/MOF-derived materials	High power density, rapid charging	EDLC/pseudocapacitance	50–1000 F g^−1^	Wearable sensors, fitness trackers	40
Energy storage	Lithium-ion batteries	MOF composites/MOF-derived materials	High energy density, rechargeable	Li^+^ insertion/extraction	100–300 mAh g^−1^	Smartwatches, wearable electronics	41
Energy storage	Flexible batteries	MOF-containing flexible devices	Lightweight, bendable	Electrochemical storage	Device-dependent	Flexible electronics	42
Energy harvesting	Piezoelectric generators	MOF composites	Converts strain to electricity	Piezoelectric effect	Device-dependent	Motion-powered wearables	43
Energy conversion	OPVs/solar fabrics	MOF composites/MOF-containing devices	Flexible, lightweight	Photovoltaic effect	5–20% PCE	Solar-powered wearables	44
Bioenergy	Microbial/enzymatic fuel cells	MOF composites/MOF-derived catalysts	Biodegradable, sustainable	Bioelectrochemical reactions	µW–mW range	Environmental/biomedical sensors	45
Thermal storage	PCMs	MOF composites	Thermal regulation	Latent heat storage	Material-dependent	Heating/cooling wearables	46

There are many types of energy storage particularly SCs are one available in different forms. These are simple parallel plate capacitors and concentric or coaxial fixed capacitors and flexible state capacitors. These capacitors are one-dimensional fibre capacitors like two CNT fibre electrodes that can be wrapped together. As a result, there will be two types of SCs coaxially structured SCs and their own processed fiber SCs. Graphite Elenev SCs did this by encouraging the creation of MXene products. SCs of MXene nature exhibit flexibility and hydrophilicity. The potential for energy storage has been met by integrating new LIBs. There are additional gadgets with printed solar panels in use. Six speeds are possible with both technologies. Centiwatts per square centimeter, or 98 MW. A pulse oximeter can be powered by this output. Certain rechargeable batteries need less charging than others, including lithium–sulfur (Li–S) batteries.^37^ TENG and other low-voltage equipment can be used with this line. It is feasible to power commercial glucose sensors. It can also charge Li–S batteries, is another advantage. The control voltage is 2 V. Power can be connected directly to the triboelectric nanogenerator (TENG). The control IC contains many peripheral devices like pressure sensors and power generators, a fission TENG. All these devices are integrated in a coaxial configuration with fibre optics, where the fibres have increased intelligence compared to other designs.

Applications of wearable devices are widely used in medical monitoring, intelligent diagnosis and analysis equipment (such as echocardiograph), internet consumer electronics industry interactive TV/STB control industrial production line edge finger inspection fault assessment infrastructure manual detection progress. Diagnostic methods and designation tools such as retinal electrical stimulation, hearing aids, walking aids, smart devices for wireless transmission, machine capsule positioning control systems and adult robots with operating arms. Consumer electronics are products that use low voltage microprocessors to run or process a high level of complexity, such as smartwatches and also include items like smart-bracelet, smartphone wireless charging devices, battery packs for cellphones and intelligent backpack.^38^ This covers military defence applications to self-power enabling gear for special troops in the field. Wireless sensor networks are utilized to monitor civil engineering projects like railroads and bridges, while Google Glass is used for failure detection in industrial settings and RFID tags track the movement of products. Wearable technology is used in the consumer electronics sector, military defence automation equipment, industrial production lines, video monitoring systems, medical detection, smart diagnostic instruments and manual assessment. Adherence to tertiary care medical testing and diagnostic equipment ranges from retinal electrical stimulation for treatment, hearing aids, whole body wirelessly powered health monitoring sensor waistbands and a device assisting in the ease of rehabilitation as well as timekeeping smartwatches; these include devices that communicate with remote capsule endoscope locating control. There are numerous types of smartwatches and bands of smartphones wireless charging suitcases in consumer electronics.^39^

### 5.1. Supercapacitor

Many electronic materials are used in SCs, including electronic polymers, MOFs, transition metal hydroxides, transition metal oxides and transition metal MXene.^43^ Among these are porous inorganic–organic hybrid materials, such as MOFs and porous coordination polymers (PCPs). Organic ligands that create bonds between transition metals and oxygen or nitrogen are used to create these MOFs. The majority of ligands have several carbon atoms as well as heteroatoms like nitrogen and phosphorus. MOFs come in a variety of forms depending on how their organic and inorganic constituents are structured. The design of these MOFs uses organic ligands to form bonds between transition metals and oxygen/nitrogen. Most ligands contain heteroatoms like phosphorus and nitrogen and many carbon atoms. Depending on the structure of the inorganic and organic components, MOFs can be of different types. The first group consists of pure MOFs, which store energy through the physical adsorption process, electrolyte ions are adsorbed on the MOF or through redox changes occurring on the metal surface. Electrodes based on metal oxides generated from MOFs are present in the second layer. These electrodes exchange electrolyte ions with one another to store energy. In general, the intrinsic electrical conductivity of both pure MOF electrodes and inorganic metal oxides generated from MOFs is poor. By developing hybrid electrodes with extra electrical characteristics, the problem can be resolved, and the conductivity can be raised overall. MOF composites offer a variety of advantages over single components, including enhanced performance, distinct chemical and physical properties and more. The last group includes carbon monoxide produced during the pyrolysis of MOF templates. Because of their large surface area, large pores and strong electrical properties, these carbons produced from MOFs are perfect for storage in EDLC at a reasonable cost. Because of these characteristics, they can offer more energy and capacity than carbon monoxide, which is present in many organic sources such as animal bones, banana peels and human hair.^44^ This depends on several elements, including the kind of substance, density, crystallinity, chemical composition, structural properties and activation (including procedure, type of reaction and activator). Table 5 depicts the overview of MOF categorization.

**Table 5:** Overview of metal–organic frameworks categorization

MOF category	Electrode material	Nanostructure	Surface area (m^2^ g^−1^)	Capacitance	Electrolyte	Electrode cycle stability	Device flexibility	References
Pristine MOF electrode	Ni-MOF	Accordion superstructure	118.42	988 F g^−1^ at 1.4 A g^−1^	3 M KOH	96.6%/5000 cycles	ASC retention of 100% up to 180° bending	47
	Cu-MOF	Layered structure	150.76	1024 F g^−1^ at 1 A g^−1^	1 M KOH	95%/6000 cycles	Retains 95% capacitance after 1000 bends	48
	Zn-MOF	Nanorod arrays	145.22	855 F g^−1^ at 2 A g^−1^	1 M H_2_SO_4_	92%/4000 cycles	No fading up to 150° bending	49
	Fe-MOF	Porous hexagonal structure	280.19	765 F g^−1^ at 0.5 A g^−1^	6 M KOH	97%/4500 cycles	Retains 90% capacitance after 2000 bends	50
	Co-MOF	Honeycomb-like structure	112.50	690 F g^−1^ at 1 A g^−1^	1 M Na_2_SO_4_	93%/5000 cycles	95% retention after 180° bending	51
	Mg-MOF	Porous cube structure	185.67	910 F g^−1^ at 2 A g^−1^	2 M NaOH	89%/3000 cycles	Retains 85% capacitance after 120° bending	52
MOF carbon electrode	Carbon	3D interconnected hierarchical sponge	117.42	369 F g^−1^ at 10 mV s^−1^	6 M KOH	93%/10 000 cycles	96% retention rate for SSC after 2000 bends	53
	N-doped carbon	Porous structure	1356	369 F g^−1^ at 10 mV s^−1^	6 M KOH	93%/10 000 cycles	96% retention rate for SSC after 2000 bends	54
	N-doped carbon bubbles	Porous bubbles	905	366 mF cm^−2^ at 1 mA cm^−2^	427 F g^−1^ at 0.5 A g^−1^	98%/10 000 cycles	SSC – 100% retention for 180° bending	55
	N-doped carbon	Porous fibrous structure	481.9	427 F g^−1^ at 0.5 A g^−1^	1 M H_2_SO_4_	—	SSC – little capacitance fading up to 180° bending	56
	Carbon nanotubes (CNTs)	Aligned tubular structure	1345	420 F g^−1^ at 1 A g^−1^	6 M KOH	95%/8000 cycles	Retains 92% after 150° bending	57
	N-doped graphene	Porous sheet structure	1200	500 F g^−1^ at 1 A g^−1^	1 M KOH	94%/7000 cycles	Maintains 94% capacitance after 1000 bends	58
	Carbon aerogel	3D porous network	890	460 F g^−1^ at 0.5 A g^−1^	2 M Na_2_SO_4_	90%/6000 cycles	90% retention after 180° bending	59
MOF/redox polymer hybrid electrode	PANI/UiO-66	Interpenetrating network structure	272.82	1015 F g^−1^ at 1 A g^−1^	PVA/H_2_SO_4_	84%/3500 cycles	SSC – 90% retention after 800 bending cycles	60
	PANI/MIL-101	Interpenetrating network structure	—	1197 F g^−1^ at 1 A g^−1^	PVA/H_2_SO_4_	96%/1000 cycles	SSC – 90% retention after 1000 bending cycles	61
	UiO-66/polypyrrole	Nanoparticle	125	90 F g^−1^ at 5 mV s^−1^	3 M KCl	96%/1000 cycles	90% retention in SSC after 1000 bending cycles	62
	ZIF-PPy	MOF particles dispersed over interconnected PPy tubes	1545.2	554.4 F g^−1^ at 0.5 A g^−1^	1 M Na_2_SO_4_	SSC – 90% retention after 1000 bending cycles		63
	PANI ZIF-67	Interweaved MOF crystals with PANI chains	73	2146 mF cm^−2^ at 10 mV s^−1^	3 M KCl	—	SSC – 90% retention after 1000 bending cycles	64
	PPy@MIL-88B	Core–shell nanostructure	145.2	610 F g^−1^ at 1 A g^−1^	PVA/H_2_SO_4_	92%/5000 cycles	Maintains 90% capacitance after 2000 bends	65
	PEDOT@UiO-67	Nanowire arrays	312.5	550 F g^−1^ at 1 A g^−1^	1 M Na_2_SO_4_	94%/4500 cycles	93% retention after 150° bending	66
MOF-derived metal/metal-oxide hybrid electrode	Co_3_O_4_@Co-MOF	Leaf-like Co-MOF sheets	453.3	1020 F g^−1^ at 0.5 A g^−1^	3 M KOH	96.7%/5000 cycles	99.7% retention for ASC following 400 bends	67
	MoO_2_@Cu@C	Quasi-circular particles with interconnected small sheets	183.27	28.27 mAh g^−1^ at 5 mV s^−1^	2 M KOH	—	SSC – No capacitance fading up to 180° bending	68
	MnO_*x*_ – MHCF	Nanoflowers on nanocubes	818	—	2 M KOH	ASC – No capacitance fading up to 180° bending		69
	MnO_*x*_ – MHCF	Spindle-like nanostructures	116.5	1232.4 mF cm^−2^ at 2 mA cm^−2^	1 M Na_2_SO_4_	97.6%/4000 cycles	ASC – 97.1% retention after 4000 bends	70
	NiCo_2_O_4_	Hollow and porous nanowall arrays	11.6	1055.3 F g^−1^ at 2.5 mA cm^−2^	2 M KOH	97.6%/4000 cycles	ASC – 86.7% retention after 20 000 bends	71
	Fe_2_O_3_@MOF	Nanocube morphology	552.8	940 F g^−1^ at 1 A g^−1^	3 M KOH	91%/6000 cycles	90% retention after 180° bending	72
	ZnO@MOF	Core–shell structure	245.7	830 F g^−1^ at 0.5 A g^−1^	1 M Na_2_SO_4_	93%/5000 cycles	Maintains 92% capacitance after 1000 bends	73
	CuO@MOF	Nanoporous structure	156.4	700 F g^−1^ at 1 A g^−1^	6 M KOH	89%/4000 cycles	Retains 85% capacitance after 120° bending	74
	MnO_2_@MOF	Nano-leaf morphology	345.3	1100 F g^−1^ at 0.5 A g^−1^	2 M KOH	95%/5500 cycles	Maintains 95% after 150° bending	75
MOF/nanocarbon/redox polymer hybrid electrode	CNTS/MOF/PANI	Core/shell structure	—	342.5 F g^−1^ at 1 A g^−1^	3 M KCl	—	Optical image of flexibility and compressibility shown	76
	UiO-66/GO/PEDOT	Porous structure with interconnected PEDOT	950	128 mF cm^−2^ at 10 mV s^−1^	3 M KCl	—	SSC – No capacitance fading on various bending and twisting conditions	77
	PANI@CNT/MOF	Core–shell structure with CNT/MOF hybrid	175.4	530 F g^−1^ at 0.5 A g^−1^	2 M Na_2_SO_4_	91%/5000 cycles	Retains 90% capacitance after 180° bending	78
	MOF@graphene	Layered 2D structure	225.3	600 F g^−1^ at 1 A g^−1^	1 M Na_2_SO_4_	92%/4500 cycles	Maintains 90% capacitance after 2000 bends	79
	ZIF-67@graphene/PANI	Interwoven structure	314.7	620 F g^−1^ at 1 A g^−1^	3 M KOH	93%/6000 cycles	Maintains 92% capacitance after 1000 bends	80
	MOF@RGO	Core–shell structure with RGO	185.6	650 F g^−1^ at 0.5 A g^−1^	2 M KOH	94%/5000 cycles	Maintains 94% capacitance after 120° bending	81
	MOF/graphene/PPy	Layered 3D porous structure	205.8	700 F g^−1^ at 1 A g^−1^	1 M Na_2_SO_4_	95%/4000 cycles	Maintains 95% after 150° bending	82

### 5.2. Metal–organic frameworks for solid-state batteries

**Fig. 4:**
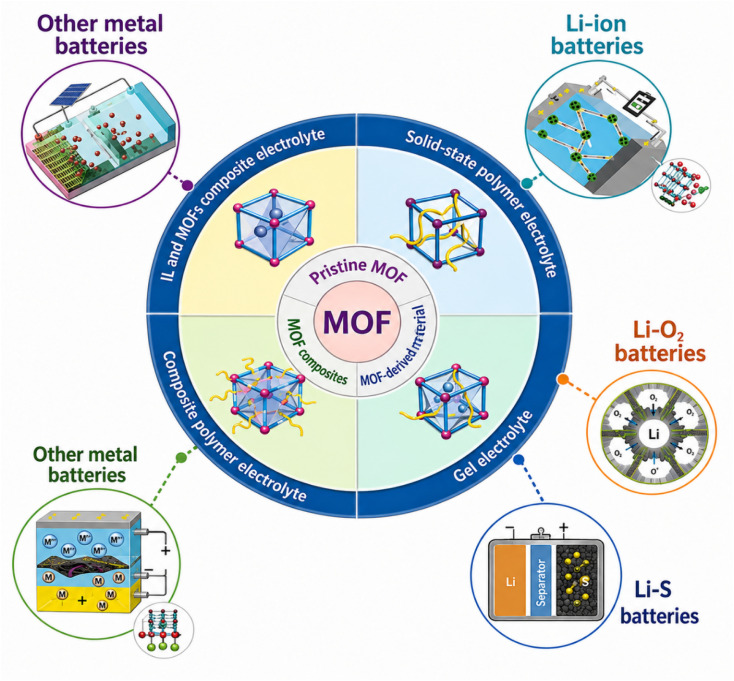
Overview of metal–organic frameworks in different types of batteries.

MOFs batteries are considered pioneers and prototypes to produce porous materials with controlled structure, low specific surface area, *etc.* Reactive sites are considered energy sources for many applications. However, the porous structure produced by the MOF derivation method is mostly powder.^40^ This requires polymer binders and conductive additives in the electrochemical reaction, which leads to fewer active sites, loss of active material, high interfacial resistance and less flexibility.^41^Fig. 4 shows the applications of MOFs in different types of batteries. Better electrical connections can be achieved by growing MOF-derived active materials directly on conductive substrates without using adhesives; this is a good way to compensate for the inadequacy. There are no reports on the corrosion of ARBs associated with the growth of MOF-derived battery materials, which form pits in existing device products and therefore become good candidates for the fabrication of battery anode materials.^42^ Doping of metal ions can improve the conductivity and electrochemical properties of battery materials, like 3D aligned NiZnCoP nanosheet arrays supported by CNT fibres as a binder-free cathode material based on MOFs.

With the development of people and the increase in population people's need for energy continues to increase. In recent years, renewable energies such as solar power and wind power have been developed extensively but the efficiency of converting the energy to useful power is low.^45–47^ Hence, the energy demand, which indicates the necessity of electricity for production and throughout the day implies that people require safe, secure, sustainable and friendly energy. Lithium batteries are widely used in many products like electric vehicles and mobile phones due to their theoretically high energy density (3860 Wh kg^−1^). They offer great benefits such as reduced weight, small size and low environmental impact. Lithium-ion batteries use liquid electrolytes (LE). Concentration changes and battery polarization due to differences in lithium-ion loading can cause uneven lithium-ion accumulation during charging and discharging. This can cause lithium dendrite proliferation, which can lead to short circuits, reduced capacitance, leakage, explosion and other safety issues. To solve these problems, researchers have used different strategies. Graphene film is used to reinforce the lithium metal anode to prevent the electrode from bending and forming lithium dendrites. Efforts have been made to develop an aluminium-lithium alloy cathode to prevent lithium dendrites from growing. However, it should be noted that these measures do not solve the current problems of lithium batteries. Solid-state batteries (SSB) are a new method.^48^ SSB is impermeable and heat resistant. The combination of these products consists of solid-state electronics and solid-state electronics (SSE), which provide excellent stability. Most importantly, the SSE can prevent lithium dendrite formation, reduce short battery life and improve battery stability. More importantly, SSE can prevent lithium dendrite growth and increase the power of lithium batteries. MOFs have many advantages such first, high porosity, large specific area, excellent kinetics and good reactivity make them reach other products, second, MOFs have high polarity, which allows them to control Lewis acid–base interaction in the body and improve their electrical properties. In addition, their porosity and specific surface area increase their surface energy, allowing them to adsorb external chemicals and foreign substances. MOFs many architectures enable them to be easily integrated with a variety of electronic devices, allowing them to exploit their superior electrical qualities to replace SSE. It can enhance SSE ionic conductivity, ion transport and other electrical characteristics. Furthermore, MOFs can be used in ion-assisted solvothermal transformation or pyrolysis to create MOF-derived chemicals. Apart from these variables, it is simple to regulate the pore size and pore type of MOFs. MOFs can improve cation exchange, offer ion shielding and increase ion migration uniformity.^49^

#### 5.2.1. Li-based batteries

**Fig. 5:**
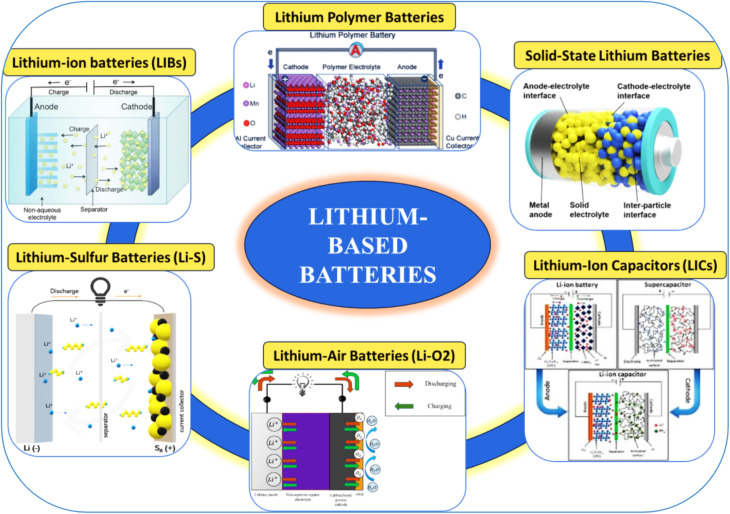
Application of metal–organic frameworks in lithium-based batteries.

Lithium-based batteries are widely available and have a lot of potential applications in electronic devices and electric cars. Liquid LIBs that offer the benefits of recycling and discharge as well as huge capacity are the most used type of lithium batteries. Lithium salt is typically used as the electrode material for the release of ions from deintercalation, organic liquid as the electrolyte material and graphite carbon as the negative electrode.^50^ But as conventional lithium-ion batteries have evolved new issues have emerged such as outdated batteries insufficient capacity to satisfy consumer demands. Lithium dendrites, which are prone to separation, can arise because of unequal lithium-ion transport. Cell polarization and variations in concentration gradients are the causes of this. In addition, other issues could arise, such as the battery short-circuiting, leaking, or even blowing up. Inadequate capacitance and lithium dendrite growth are two issues that have been researched in various ways. These include using isolation switching, additional electrical devices and electrode protection.^51^Fig. 5 shows the different applications of MOFs in lithium-based batteries.

Lithium is released from the battery electrolyte during charging and discharging, causing cracks to form at the SSE interface. Therefore, side reactions will occur between the liquid electrolyte and lithium metal. Consequently, the issue of lithium dendrite formation in liquid electrolyte systems cannot be resolved by this approach alone. Simultaneously, to enhance the solid-state electrolytes interaction with the electrodes, researchers are attempting to develop solid-state lithium batteries.^52^ These batteries provide a lot of benefits, like increased stability, high mechanical elasticity non-volatility and non-flammability. They cannot, however, transport enough ions. A lot of focus has been placed on the special qualities of MOF materials, like high ductility and elevated metal activity, to get beyond these restrictions. To enhance the electrochemical action of solid-state lithium batteries, MOFs have been added.

#### 5.2.2. Lithium-ion batteries

**Fig. 6:**
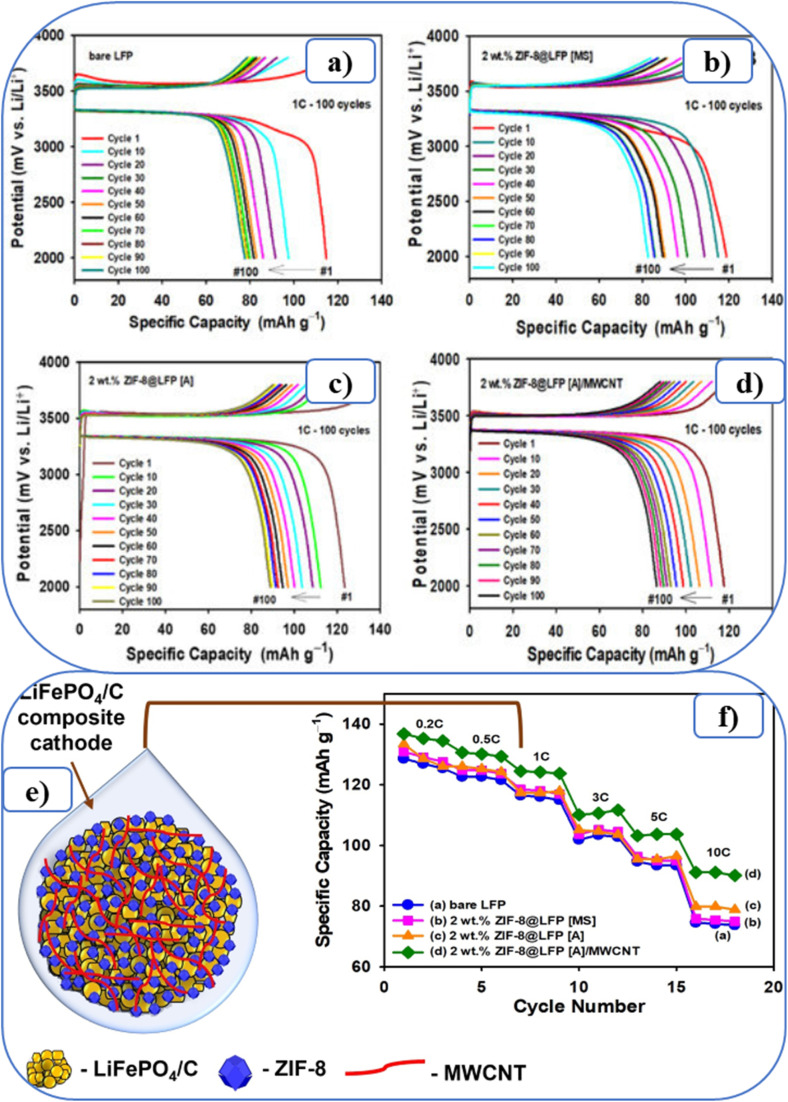
(a) Charge–discharge curves of bare LFP, (b) 2 wt% ZIF-8@LFP [MS], (c) 2 wt% ZIF-8@LFP, (d) 2 wt% ZIF-8@LFP/MWCNT electrodes at 1C/1C rate for 100 cycles, (e) schematic representation of the synthesis of ZIF-8 and mechanofusion-assisted MWCNT Coating of LiFePO_4_/C, and (f) specific capacity analysis of different samples. Reproduced from ref. 53 with permission from MDPI, copyright 2023.

Large machines and numerous mobile electronic devices frequently employ LIBs as their primary battery technology.^53^ Numerous benefits come with lithium-ion batteries including their ability to function at high temperatures, huge storage capacities, memory lessness, lightweight construction and environmental friendliness. In Fig. 6, Li^+^ ions travel from the polymer separator to the positive or negative electrode during charging and discharging. But at the start of the stripping process, the pure lithium electrode created a unique and permeable lithium metal structure, which caused the subsequent lithium plating process to produce lithium dendrites and this problem can be resolved by adding MOF. MOFs enhance lithium-ion battery performance through two primary ways.^54^ The first step is to create an ion screen. By incorporating MOFs with large pore sizes and strong cationic sites into solid-state structures (SSEs), the movement of anions can be effectively restricted. The particularly large surface area of MOFs facilitates effective interaction with Li^+^ ions and other materials, reducing the energy required for transport and interlayer formation. This leads to a uniform distribution of Li^+^ ions during electrodeposition.^55^ Due to their large pore size, MOFs can serve as screening agents. During the preparation of MOFs, reaction conditions can be controlled to produce MOFs with large pore sizes. This large pore can be used to selectively absorb different anions, thereby enhancing the electrical properties of lithium ions and improving the electrical properties of the battery. Anions can also be immobilized using other techniques.^56^ Because of their special structure, MOFs can use adsorption or electrostatic forces to immobilize anions in the electrolyte. The immobilization process gives lithium ions a reliable and effective route, enabling unrestricted movement. Binding anions to a neutral metal without saturation is a popular technique for immobilizing anions. Immobilized anions in lithium-ion batteries efficiently divide the anions from the Li^+^ ions. The electrolyte's crystalline structure is lowered during this process, which also makes Li^+^ ions travel more quickly.^57^

Because of its affordability and friendly to the environment, LiFePO_4_ constitutes one of the commercial, adaptable cathode materials used in the fabrication of lithium-ion batteries. Despite its inadequate ionic and electron conductivity, LiFePO_4_'s electrochemical efficiency during higher current rate performance is still constrained. The present study produced a composite cathode material made of LiFePO_4_/C (LFP) modified by multi-walled carbon nanotubes (MWCNT) and zeolitic imidazolate framework (ZIF-8). When the ZIF-8 coating was treated by the agitator, the LFP composites homogeneous as well as stable spherical morphology was achieved. The LFP composite cathode materials improved lithium-ion transport characteristics, reduced polarization impact, and interfacial impedance all of which came from the combined synergistic effect of the ZIF-8 and MWCNT coating materials.^57^Fig. 6(a–d) shows the charge–discharge curves of different composites at 1C/1C rate for 100 cycles, Fig. 6(e) is the schematic representation of the synthesis of ZIF-8 and mechanofusion-assisted MWCNT coating of LiFePO_4_/C, and (f) specific capacity analysis of different samples.

An interesting and novel way to solve the difficulty of lithium dendrite formation in lithium batteries is to use ion sieves for modification. Han *et a*l. recently developed an electrolyte called MOFs-SN-FEC using a high-temperature impregnation method of MOFs.^58^ The space in MOFs is used to act as a filter for ions in GPE (gel polymer electrolyte). This not only restricts the movement of other large ions but also promotes the movement of Li^+^ ions, providing a more uniform distribution, thus preventing lithium dendrite formation due to uneven transport. At the same time, the electrolytes were also tested for lithium-ion conductivity. The study result determined it as 7.04 × 10 × 4 S cm × 1, which is better than the behaviour of unmodified MOF material. Furthermore, the electrochemical performance of MOFs-SN-FEC electrolytes in LIB was also evaluated. The test results show that after 100 cycles of the battery at 0.1C rate, the capacity retention rate is still 98.8%. The interfacial resistance and charge transfer of LIB using MOFs-SN-FEC were measured as 368 Ω. The battery performs well at different rates. Also, no lithium dendrites were formed during the operation. This provides additional evidence of the safety and security of the battery.^58^

#### 5.2.3. Lithium–sulfur batteries

**Fig. 7:**
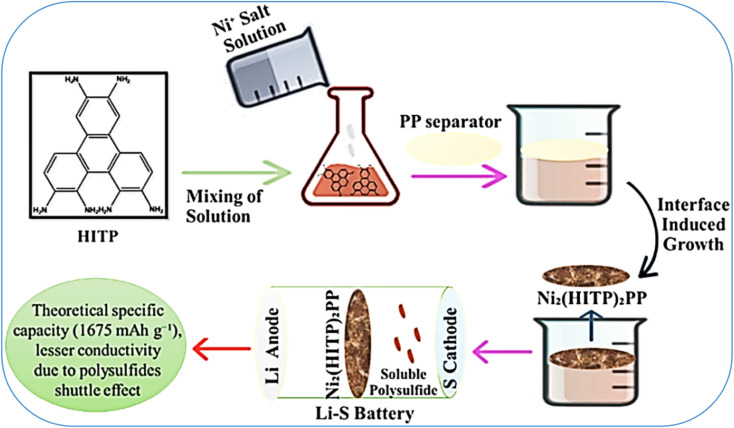
Synthesis of metal–organic frameworks for lithium–sulfur batteries application. Reproduced from ref. 59 with permission from RSC, copyright 2024.

Li–S batteries have many advantages over other lithium batteries, including higher energy consumption, lower cost, less self-discharge and improved battery power conversion. However, it is important to recognize the shortcomings of lithium–sulfur batteries, such as the poor electrical properties of sulfur and the formation of macromolecular lithium polysulfide (LiPS) intermediates during cycling.^59^ These intermediates tend to shuttle between electrode tips and accumulate on their surfaces. To solve these problems, researchers are trying to incorporate SSE into lithium–sulfur batteries. SSEs can effectively affect the separation of LiPS intermediates and prevent their shuttle effect. Furthermore, the use of special structures and functionality of MOF materials can improve the electrical properties of Li–S batteries. Fig. 7 shows the schematic illustration of various steps for the synthesis of MOFs for Li–S batteries application.

A promising way to achieve this goal is to focus on electrodes, mainly the use of sulfur-based cathodes. These substrates usually need to be able to attract and store sulfur molecules by physical or chemical means. This ensures that the LiPS produced is properly captured during battery life, preventing the shuttle effect. MOFs are good matrix materials because of their porosity and ability to control size. The pore size of the MOF is an important factor. Increasing the pore size allows more sulfur loading while decreasing the pore size prevents the movement of more sulfur species. According to experimental findings, particles with a size of about 200 nm can efficiently catch LiPS and lessen the shuttle effect. Although solid MOFs perform poorly in batteries, this issue can be resolved by mixing MOF with other high-energy materials to create MOF derivatives and composites that have very high yields. As an alternative, lithium anodes in lithium–sulfur batteries may be stabilized by developing potential electrolytes. Although lithium anodes have a large theoretical capacity, stripping and back-coating cause them to slowly degrade over time.^60^

**Fig. 8:**
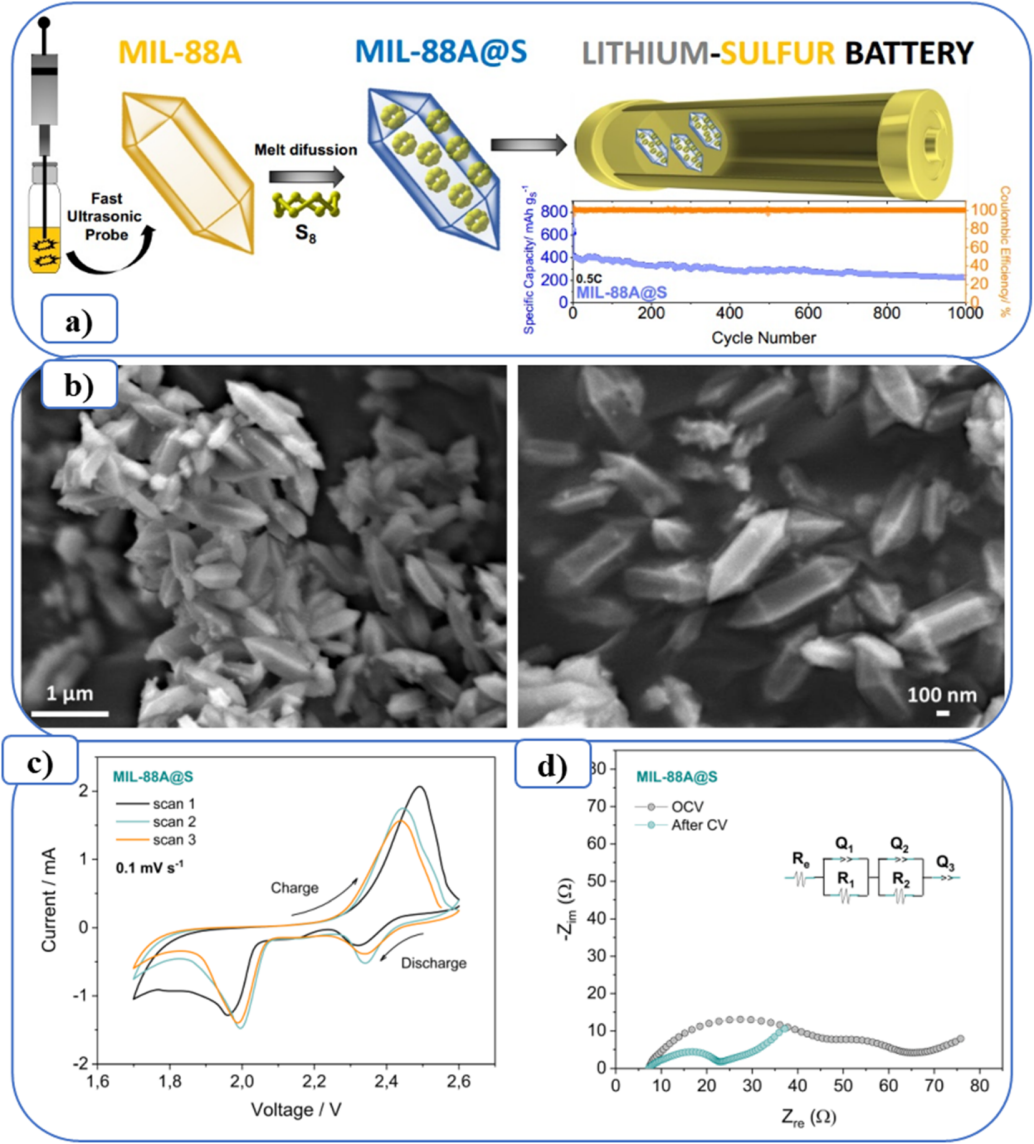
(a) Schematic representation of the synthesis of MIL-88A metal–organic framework as a stable sulfur–host cathode, (b) SEM images, (c) CV profiles of Li/LiTFSI-LiNO_3_-DOL: DME/MIL-88A@S battery and (d) EIS measurements. Reproduced from ref. 58 with permission from MDPI, copyright 2020.

Furthermore, the shuttle effect of LiPS affects the electrolytes stability. One benefit of SSE over traditional liquid electrolytes is their lower size. As a result, the anode has greater room, improving the battery's energy density. Electrolytes combined with MOFs exhibit improved ion selectivity. Metal ions in the MOF provide more neutral coordination sites for Lewis bases. In addition, MOFs effectively immobilize many anions from the protective surface, reducing their effects on the electrolyte. Regulation of anion transport also regulates the movement of lithium ions and other anions. By preventing the formation of charged sites, lithium dendrites can be prevented from growing, thereby improving the stability of the lithium anode and improving the electrochemical action of the lithium–sulfur battery.^61^ In addition to the low price, excellent density of energy as well as sustainability, Li–S batteries have drawn a lot of interest as a potential energy storage technology to rival finite, non-renewable energy sources. However, it is the “shuttle effect” that causes the active material to corrode and lose its strength while cycling. To address this problem, MOF are thought to be superior sulfur host materials that can effectively contain unwanted polysulfides when used as a cathode in lithium–sulfur batteries. Utilizing iron fumarate, MIL-88A has been produced using a quick and easy ultrasonic-assisted probe technique. And show remarkable coulombic efficiency. They also maintain a reversible capacity exceeding 300 mAh g^−1^ around 0.5C for 1000 cycles, making them a useful cathode constituent during prolonged cycling in Li–S cells.^62^Fig. 8 shows the (a) schematic representation of the synthesis of MIL-88A metal–organic framework as a stable sulfur-host cathode, (b) SEM images, (c) CV profiles of Li/LiTFSI-LiNO3-DOL: DME/MIL-88A@S battery and (d) EIS measurements. Aluminium terephthalate metal–organic framework (Al-TPA-MOF) was prepared by electrolysis process. Researchers have developed MOFs as additional materials to enhance the performance of composite polymer electrolytes in lithium–sulfur (Li–S) batteries. The electronic components synthesized using this structure exhibit excellent thermal stability and remain stable up to a temperature of 270 °C.^57^ Analysis of the ionic conductivity of the electrolyte shows that the conductivity is highest when the composite contains 10% Al-TPA-MOF. These electrolytes are used to construct all-solid-state lithium–sulfur polymer batteries.^62^Table 6 shows various applications of flexible MOFs in solid-state batteries.

**Table 6:** Flexible metal–organic frameworks in solid-state batteries

Battery type	MOF category	Material combination used	Nanostructure	Surface area (m^2^ g^−1^)	Capacitance (F g^−1^)	Electrolyte	Cycle stability (%)	Device flexibility	References
Lithium-ion batteries	MIL-101 (Cr)	Cr-based MOF with carbon	Nanosheets	1500	250	LiPF_6_ in EC (1 : 1)	90	High	83
	UiO-66 (Zr)	Zr-based MOF with graphene	Nanoparticles	1200	220	LiBF_4_ in PC	85	Moderate	84
	MOF-74 (Zn)	Zn-based MOF with a polymer blend	Nanofibers	1400	200	LiTFSI in DMSO	80	High	85
	ZIF-8 (Zn)	Zn-based MOF with silica	Nanosheets	1350	210	LiCl in EC	75	Moderate	86
	MIL-125 (Ti)	Ti-based MOF with carbon black	1D nanostructures	1300	230	LiPF_6_ in EC	88	High	87
	HKUST-1 (Cu)	Cu-based MOF with conductive polymer	Nanoplates	1250	240	LiNO_3_ in DMSO	78	Moderate	88
	ZIF-67 (Co)	Co-based MOF with organic compound	Nanospheres	1450	250	LiTFSI in DME	82	High	89
Li–S batteries	MIL-53 (Al)	Al-based MOF with sulfur	Nanowires	1300	150	LiTFSI in DMSO	75	Moderate	90
	ZIF-8 (Zn)	Zn-based MOF with S-doped carbon	Nanosheets	1400	160	LiPF_6_ in EC	72	Low	91
	UiO-66-NH_2_ (Zr)	NH_2_-functionalized Zr-based MOF	Nanofibers	1150	170	LiNO_3_ in DMSO	78	Moderate	92
	MIL-125 (Ti)	Ti-based MOF with sulfur	Nanoparticles	1200	155	LiCl in EC	76	Moderate	93
	HKUST-1 (Cu)	Cu-based MOF with sulfur	2D nanosheets	1250	145	LiPF_6_ in DME	70	Low	94
	MIL-101 (Cr)	Cr-based MOF with S-doped carbon	Nanospheres	1350	150	LiBF_4_ in PC	74	Moderate	95
	ZIF-67 (Co)	Co-based MOF with polysulfides	Nanofibers	1200	155	LiTFSI in DMSO	68	Low	96
Zinc-based batteries	MOF-5 (Zn)	Zn-based MOF with carbon fibre	Nanowires	1350	220	ZnSO_4_ in H2O	82	High	97
	ZIF-8 (Zn)	Zn-based MOF with a polymer blend	Nanosheets	1500	240	ZnCl_2_ in EC	80	High	98
	MIL-53 (Fe)	Fe-based MOF with carbon	Nanofibers	1450	210	ZnSO_4_ in H2O	78	Moderate	99
	UiO-66 (Zr)	Zr-based MOF with graphene oxide	Nanoparticles	1400	230	ZnTFSI in EC	85	High	100
	ZIF-67 (Co)	Co-based MOF with S-doped carbon	1D nanostructures	1380	200	ZnCl_2_ in EC	76	Moderate	101
	MOF-74 (Mg)	Mg-based MOF with conductive polymer	Nanoparticles	1300	215	ZnSO_4_ in H2O	80	Moderate	102
	MIL-125 (Ti)	Ti-based MOF with ZnO	2D nanosheets	1400	175	ZnTFSI in EC	75	Low	103
Sodium-based batteries	MIL-101 (Cr)	Cr-based MOF with sodium ions	Nanosheets	1100	190	NaCl in H2O	80	Moderate	104
	ZIF-8 (Zn)	Zn-based MOF with Na_2_SO_4_	Nanospheres	1300	185	NaNO_3_ in EC	78	Low	105
	MOF-74 (Mg)	Mg-based MOF with sodium ions	Nanofibers	1200	200	Na_2_SO_4_ in DMSO	82	Moderate	106
	UiO-66 (Zr)	Zr-based MOF with sodium salts	Nanoparticles	1150	195	NaTFSI in DMSO	81	Moderate	107
	MIL-125 (Ti)	Ti-based MOF with NaPF_6_	2D nanosheets	1400	175	NaCl in DMSO	75	Low	108
	MIL-53 (Fe)	Fe-based MOF with sodium	Nanowires	1300	190	NaNO_3_ in EC	70	Moderate	109
	ZIF-67 (Co)	Co-based MOF with sodium salt	Nanosheets	1200	185	Na_2_SO_4_ in DMSO	73	Low	110

#### 5.2.4. Zinc-based batteries

When lithium-based batteries are compared with zinc-based batteries, the latter have the advantages of lower cost, safety and less pollution. However, zinc-based batteries also have the disadvantages of slow transport and low cycling, which affect their wide application. The main function of SSEs containing MOFs is to immobilize anions. These MOFs contain many pores that can absorb and trap anions through Lewi's acid–base interactions, thus promoting the movement of zinc ions. In addition, SSEs have a good relationship with zinc anodes. A special nano-wet interface can be created between the two materials, which improves zinc deposition and ensures a uniform and thick zinc layer during deposition. This prevents the formation of zinc dendrites that can penetrate components and short-circuit the battery. MOFs can also be very effective as cathode catalysts in zinc-air batteries. They use porous structures to enhance the oxygen reduction reaction (ORR) and oxygen evolution reaction (OER) pathways. This increases the transport of intermediates, thus increasing the property of OER and ORR. Therefore, the electrochemical performance of the battery is improved.^92^

**Fig. 9:**
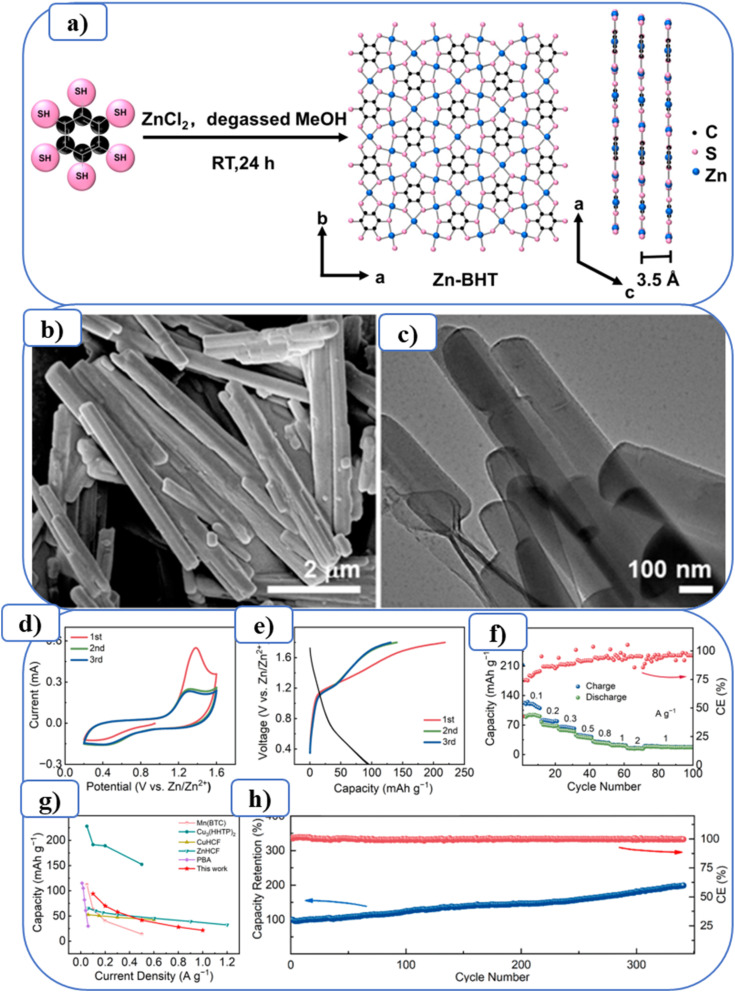
(a) Synthesis process of 2D Zn-BHT MOF, (b) SEM, (c) TEM of Zn-BHT microrods, electrochemical properties of Zn-BHT. (d) CV curves, (e) GCD, (f) rate performance, (g) comparison of specific capacities, and (h) cyclic stability. Reproduced from ref. 88 with permission from MDPI, copyright 2024.

High-potential electrochemical energy storage (EES) devices known as zinc-ion capacitors (ZICs) combine the benefits of high-power SCs with high-energy zinc-ion batteries. The development of ZIC is still significantly hampered, though, by the lack of appropriate cathode materials with robust crystal structures and many channels for stable and quick Zn^2+^ ion transport. The electrochemical performance of zinc-benzenehexathiolate (Zn-BHT), 2D MOF material with stable and conductive characteristics, with unique structure of Zn-BHT led to good cycle performance, advantageous rate capability, and a high reversible discharge capacity of 90.4 mAh g^−1^ at 0.1 A g^−1^.^93^Fig. 9(a) shows the synthesis process of 2D Zn-BHT MOF, (b) SEM, (c) TEM of Zn-BHT microrods, Electrochemical properties of Zn-BHT. (d) CV curves, (e) GCD, (f) rate performance, (g) comparison of specific capacities, and (h) cyclic stability. Reproduced with permission from.^93^ Modern zinc–air batteries suffer from slow ORR and OER, causing significant problems for researchers. So far, adding catalysts has been proven to be an effective treatment. Chen *et al.* Using ultrathin MnO_2_ hollow nanowires as templates, porous MnO@Co-N/C nanomaterials were prepared by a series of steps including hydrothermal method and thermal cracking. After the test, they found that MnO@Co-N/C exhibited the highest limiting current of 5.52 mA cm^−2^. Compared with other samples, the MOF composite sample exhibited excellent ORR catalytic activity, when measuring the OER catalytic performance, the rotation speed was set to 1600 rpm.^94^

#### 5.2.5. Sodium-based batteries

**Fig. 10:**
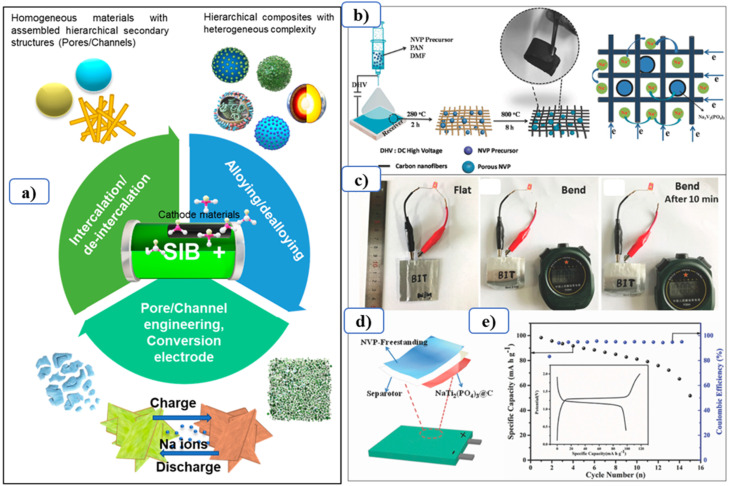
(a) Various steps for NVP-freestanding composite material preparation. (b) Electronic and Na^+^ diffusion pathways, (c) digital images showcasing the sodium-ion soft package battery, (d) internal structure of flexible sodium-ion soft package battery, and (e) electrochemical studies. Reproduced from ref. 91 with permission from MDPI, copyright 2024.

The operation of sodium batteries is like lithium batteries. However, sodium batteries always face safety issues due to the use of liquid electrolytes, such as electrolyte instability and evaporation, which will cause the formation of unstable sodium dendrites. Therefore, the use of SSE has become a trend in the development of sodium batteries.^95^ MOFs can use their unique structures to store and selectively filter different ions, as well as promote the transport of sodium ions and limit the movement of large anions. MOFs can be effectively used in sodium-based batteries by controlling their different structures, such as the pancake type (which produces more energy) and the red battery type (which promotes oxygen exchange and causes great stress).^96^ MOFs are suitable for working with specific groups, allowing for special combinations of effects. For example, they have high electronegativity, which increases the density of the surrounding air and increases the resistance of objects with more electrons. This reduces the diffusion capacity of Na^+^ ions, allowing them to move faster. This functional group also plays an important role in improving the performance of sodium-based batteries. Compared to LIBs, SIBs have a lower energy density and poorer cycle stability. Sodium vanadium phosphates (NVPs) and sodium vanadium fluorophosphate (NVPFs) are two of the least expensive cathodes with high potential capacities in SIBs. These cathode materials have a short cycle life and poor rate performance due to their low conductivity. Numerous synthetic techniques have been studied to increase conductivity, such as surface modification, heteroatom doping (N, S, and Co), the creation of porous nanostructures, and the creation of composites with conductive carbons. Significant promise has been shown by cathodes with hierarchical, interconnected micro- and nanoporous morphologies using NVPs and NVPFs in carbon composites.^97^Fig. 10(a) shows the various steps for NVP-freestanding composite material preparation. (b) Electronic and Na^+^ diffusion pathways, (c) digital images showcasing the sodium-ion soft package battery, (d) internal structure of flexible sodium-ion soft package battery, and (e) electrochemical studies.

Na–S batteries exhibit a remarkably high theoretical capacity among Na-based batteries, making them highly attractive for future research endeavors. Nevertheless, the inadequate conductivity of sulfur and the sluggish reaction rate result in numerous drawbacks in its practical use. Xiao *et al.* designed and utilized a porous carbon host that was derived from MOFs and doped with S and N in Na–S batteries. This design resulted in improved electrochemical properties of the battery. The electrode demonstrated high cycle stability when combined with MOF composites. After undergoing 100 cycles at a rate of 0.1 A g^−1^, it still retained a capacity of 691 mAh g^−1^ Furthermore, it exhibited a capacitance of 90% after 500 cycles at a rate of 1 A g^−1^. Furthermore, it exhibited outstanding outcomes across various levels of electric current densities. The explanation for this improvement in stability was the combination of SSEs with sulfur, resulting in the formation of covalent sulfur and the prevention of the shuttle effect. Furthermore, this experiment showed the possible application of composites produced from MOFs in sodium batteries.^97,98^

## 6. Opportunities and emerging applications for MOFs

### 6.1. Computational screening and data-driven MOF design

MOFs are used to measure gas adsorption and diffusion in their holes.^99^ They can also be used to define the limits of gas separation and adsorption performance in membrane processes. MOFs can also be compared to conventional and commercial porous materials based on adsorbent and film performance measurements.^100^ They can also be used to relate MOF properties to their gas separation capabilities. They can also be used to identify small pieces of information provided by many MOFs.^101^ Finally, MOFs can be used to accurately analyze and find new MOF materials with desired properties and excellent gas separation by computational methods, thus demonstrating experimental work, saving time and better-allocating resources.^103^ In the molecular modelling of MOFs, various theories and methods have been used to calculate adsorbent and membrane performance metrics.^104^ While the transfer of the framework is important for MOFs that need to breathe, the overall energy may not be suitable for MOFs with Open Metal Site (OMS) but may be good for MOFs without OMS. Therefore, it is important to understand the properties of the MOFs to be evaluated, and the molecular models can be modified accordingly. Computer-generated MOFs are helping to discover more materials than experimental scientists can put together. Therefore, they play an important role in establishing relationships between properties that cannot be studied due to the limited availability of synthetic materials.^104^

Porous polymer networks (PPNs), zeolites, ZIFs, MOFs and hMOFs are some of these materials.^105^ The outcomes of molecular simulations serve as the foundation for this comparison. According to their findings, it might be difficult to reach the present Advanced Research Projects Agency-Energy (ARPA-E) aim for fossil fuel storage.^106^ The gas separation potential of various MOF-like materials should be investigated using comparable methods, particularly when it comes to applications involving the separation of carbon dioxide. Comprehensive molecular-level data on MOF and MOF composite performance can be obtained through sophisticated analytical techniques. For instance, a novel class of MOFs called multivariate (MTV)-MOFs was created utilizing various materials and/or metal nodes.^107^ The CO_2_/N_2_:10/90 selectivity was ascertained by doing a large screen of 10:995 MTV-MOFs with various connections in a single sample. The findings indicate that small-pore MOF functionalization yields better CO_2_ capacity and selectivity than large-pore MOF functionalization.

Another example involves the process of functionalizing MOFs with ionic liquids (ILs), which are salts containing cations and anions.^108^ According to the results of previous high-pressure tests, the incorporation of IL into hMOF improved the CO_2_/CH_4_ separation ability compared to the original MOF. The computational method used in molecular simulations accurately predicts the oil uptake of newly synthesized IL/CuBTC composites, which is consistent with the experiment.^109^ This enables the calculations to be transferred to various IL-conjugated CuBTC structures. Machine learning techniques are currently used to simulate the results to establish a relationship between the adsorption/separation properties and the properties of MOFs.^110^ Molecular simulations were used to develop the descriptive models and gas adsorption properties of MOFs. As mentioned earlier, these simulations are based on certain assumptions. It would be very interesting to study the effect of changing theories or molecular simulations on the results of machine learning algorithms. Most of the mechanistic studies have focused on hMOFs due to the large amount of information available in this repository, including a wide variety of connectors, metals and topologies.^111^ Although the number of data is small compared to hypothetical data, the application of machine learning to MOFs is real or designed to create opportunities. This approach allows the creation of new MOFs with similar structural and/or topological properties to existing MOFs, while at the same time providing better gas separation performance.

### 6.2 For wearable and portable electronics

Wearable electronics have made significant progress in recent years, changing the way we use technology. Wearable devices, including artificial intelligence, smart watches, health monitoring and virtual reality glasses, have become an important part of our daily lives.^112^ However, effectively integrating these devices into our daily activities requires reliable and efficient energy storage that can provide the best performance in a small and flexible design.^113^ Renewable energy, beyond non-renewable energy, accounts for 19% of all global energy production. Among all available renewable energy sources, biomass is the largest issue, accounting for 9% of world energy consumption.^114^ Different forms of renewable energy include biomass, biomass heat, ethanol, biomass diesel, wind energy, solar energy, geothermal energy, thermal energy, ocean energy, *etc.* Together, they are changing the way we currently receive and use energy.^114^

SCs can store the extra electricity produced by the incorporation of organic matter in biomass energy. SCs can be divided into three types: electric double-layer capacitors (EDLC), hybrid capacitors and pseudo capacitors. EDLC stores energy by rapidly absorbing and releasing electrolyte ions on the electrode material.^115^ These capacitors are very stable for cycling and usually have carbon-based materials known for their large-area-specific properties. Pseudocapacitors differ from conventional electronic devices in that they store charge at the interface between the electrode and the electrolyte through the reverse faradaic redox process instead of static electricity. Transition metal oxides are good choices for electrode materials because of their pseudocapacitive properties. Pseudocapacitors have a low-voltage and stable cycle due to the continuous product and controller that occur during discharge and recovery. Hybrid capacitors are at the forefront of energy storage technology. They provide a unique combination of rapid charge and large energy storage capacity by leveraging the extraordinary power of conventional capacitors and batteries. This allows them to deliver short energy pulses, which is excellent for applications requiring high power consumption Hybrid capacitors have a long service life, withstanding numerous charges and discharges with low losses. Due to their long life and ability to meet current, high-power requirements and long-term power requirements, they are well adapted to many industries, including mechanical and electrical continuity.^116^

### 6.3. 3D printing and additive manufacturing of MOFs

The 3D printing technology is one of the most compelling manufacturing technologies since it can develop complex models using moulds.^117^ This method can shape MOF powder into 3D MOF structures, thereby enhancing their applicability in industrial applications. Compared with traditional moulding methods, 3D printing offers the following advantages: (i) virtually any shape or design of specialized materials can be created using various types of modelling software, and (ii) all design data can be digitally transferred directly to the printer, enabling accurate and efficient fabrication. However, 3D printed MOF monolithic concepts made by this model generally have cross-linking, which enables heat and air transfer during operation (iii) unlike granulation, 3D printers can generate controls inside monolithic materials, which creates pressure drop or shock. (iv) Interaction and treatment of different substances in specific applications such environment by protecting the active sites of MOFs. The data are transformed into transferable information for various applications, including water purification, gas separation, drug delivery, disease diagnosis, fuel storage, humidity sensing, catalysis, batteries, and biomedical applications.^118^

### 6.4. Light-emitting diodes and optoelectronic applications

Light-emitting diodes (LEDs) are widely used in many applications such as lighting, electronics and optoelectronic devices due to their properties such as efficiency, long operating life and low energy consumption. Researchers continue to search for new materials to create LEDs with better performance and the ability to adjust the colour of their emissions.^119^ The metal–organic framework has features such as adjustable porosity, large surface area and various chemical functional groups that enhance LED performance. State-of-the-art electronic products are known for their high-performance, high-energy efficiency, environmental friendliness, long life and easy packaging, providing good illumination and being environmentally friendly for many uses. Another way to reduce the environmental damage caused by mercury leaks from overused light bulbs and fluorescent tubes is to use light-emitting diodes. There are two main methods of making such LEDs.^120^ The first used a simple set of red, green and blue (RGB) LEDs. The second is called a phosphor-switched white light emitting diode (pc-WLED), which uses chromaticity dots to coat RGB phosphors on UV LED chips or yellow phosphors on blue LED chips. Rare-earth doped materials are particularly attractive among luminescent materials because of their rich orbital configuration, low phonon energy, concentrated energy density and excellent thermal and environmental stability. Due to the transfer energy of rare-earth ions, co-doped materials exhibit excellent luminescence properties and bidirectional luminescence switching has been achieved using rare-earth dopants. With an apparent colour temperature of 4035 K and a colour rendering index of 95, this phosphor displays stunning colours.^121^ This model lowers the brightness of white light-emitting diodes (WLED) goods by combining nitrate and H_3_BTC (BTC = benzene-1,3,5-tricarboxylate). More than 90.6% of the original value can be controlled by varying the molar ratio, which will change the emission tone. Two kinds of blue phosphors are used in the production of two MOFs to create WLEDs. These two devices have been found to have CIE coordinates of (0.31, 0.33) and (0.31, 0.34), indicating that they are both capable of emitting white light.^122^

### 6.5. For energy harvesting and self-powered systems

Effective MOFs in TENGs based on zeolitic imidazole framework (ZIF), ZIF MOF is used in sensor and self-powered systems.^123,124^ Zeolitic imidazole framework-8 (ZIF-8) and Kapton were used as building blocks in the development of MOF-TENG. ZIF-8 was synthesized in solution on polyethene terephthalate (PET) and indium-doped tin oxide (ITO) substrates.^125^ It is possible to generate ZIF-8 films with varying thicknesses by varying the number of cycles. The MOF-TENG operates in a vertical splitting mode where the positive and negative triboelectric layers are ZIF-8 and Kapton, respectively.^126^ Surface potential and electrical studies verify that MOFs are suitable candidates for TENG applications. After 20 ZIF-8 development cycles, the MOF-TENG produced a stable output of 164 volts and 7 microamperes on vertical contact.^127^ The performance improvement was attributed to the increase in surface capacitance and the development of special surface structures. Additionally, MOF-TENG for low power consumption, detection of UV radiation and detection of tetracycline.^128,129^ In addition to the thickness effect, the performance of MOF-TENG is also affected by various ZIF subclasses.

ZIF and Kapton are used as triboelectric layers.^130^ Electrical tests show that ZIF-7 performs best as a material. ZIF-7TENG performs best when operating in vertical insulation with an output voltage of 60 V and an output current of 1.1 µA. The performance difference is due to the difference in roughness. Finally, the team used the energy stored in the ZIF-7 charged capacitor to power a variety of electronic devices, including timers, calculators, measuring devices and ultraviolet and infrared LED outputs. Furthermore, the performance of TENGs can be enhanced by combining MOFs with polymers. HKUST-1 and polydimethylsiloxane (PDMS) nanocomposite films were used to create a unique wet TENG.^131^ Through the addition of 5% of HKUST-1 weight, the TENG performed well. 37 microamperes of maximum output current and 205 volts of maximum output voltage are possible. Furthermore, the operating voltage of 3 MΩ is required to achieve the maximum output power of 17.10 mW, which is 13 times larger than the TENG composed solely of PDMS. More significantly, whereas composite TENGs retain or even enhance their efficacy in humid environments, traditional TENGs perform poorly.^132^ The MOFs can be added to other materials to improve TENG performance. Fluorinated MOFs (F-MOFs) were reported to be efficient materials for the fabrication of high-performance TENGs.^133^ This advanced triboelectric device acts as an air filter by using the electric charges generated by the friction of the membrane to remove airborne particulate matter (PM). The excellent surface area has excellent electrical properties and good structure.

## 7. Challenges and future scope

One of the main challenges of MOF-based energy storage is maintaining stability under heavy use. Research should be conducted to create more stable MOFs, especially in humidity, high pressure and chemical reactions. Encapsulation and protection layer technology could also be explored. Progress in supercapacitors and battery technology should leverage metal–organic framework-based electrode materials. To enhance electrical performance and reduce costs, researchers must investigate the integration of MOFs with other electronic materials or develop composite architecture. This may involve the fabrication of MOF-based electronic devices with varying compositions and porosities. Advanced *in situ* characterization techniques can elucidate the electrochemical behavior of MOF-based electronic devices for the advancement of novel materials and technologies. Despite the advantageous characteristics of MOFs, their large-scale synthesis continues to pose a hurdle. Research is needed to improve the fabrication process, increase efficiency and build production capacity for MOFs to be used in energy storage applications. Collaboration between data scientists and engineers is important in this case.^132^ Significant progress has been made in MOF research and while some MOFs have demonstrated stability and structural understanding, questions about the stability and structure of some MOFs remain unanswered. Future work will focus on uncovering the principles by which crystalline structures are destroyed over time and developing strategies to improve their stability. This will help design and fabricate MOFs with improved long-term stability and durability. Due to their extraordinary flexibility, continued research will be important to modify MOF properties to suit specific applications.^133^ By selecting appropriate metal ions, functional groups, and organic linkers, MOFs can exhibit certain characteristics. Regulating the pore size and volume of MOFs may enhance the adsorption of gases of varying dimensions in the domains of sensing and fuel storage. This enhances the selection and efficacy of fuel storage. This greatly affects environmental protection and energy storage. In the field of catalysis, selective and efficient catalytic reactions can be attained by modifying the structure of MOFs.^132^ Researchers can customize MOFs to meet the specific demands of catalytic reactions by selecting appropriate metal ions and organic ligands. Researchers can boost catalyst performance by modifying parameters such as pore size and surface functional groups, thereby improving the interaction between the environment and effective chemistry.

In the domains of biosensing and nanomedicine, it is possible to selectively change MOFs using metal ions, organic molecules, and functional groups. This alteration facilitates precise medication administration, particular biomolecule recognition, and tailored treatment. Tailored concepts enhance treatment accuracy and establish the groundwork for personalized and precision medicine. The tailored MOF structures will significantly influence research and future applications by enhancing the efficiency, precision, and control of material solutions across many domains, hence advancing scientific and technological advancement. The collaboration in MOF research has enhanced competence in chemistry, materials science, physics, and engineering, hence broadening the application scope of MOFs. This will enhance the creation of MOFs that exhibit superior performance across various applications. MOFs possess significant potential for many applications, including catalysis, supercapacitors, batteries, and energy conversion and storage.^133^ Subsequent research endeavors will centre on crafting MOFs with elevated surface area, distinct pore size and redox characteristics to enhance their efficacy across various domains. Technology for storing and converting renewable energy will therefore succeed. Because of their excellent adsorption and separation qualities, MOFs are highly appropriate for industrial and environmental applications. Future studies will concentrate on using these materials for water treatment, gas separation and carbon capture, among other environmental issues.

MOFs can also be used to increase the efficiency and stability of these processes in various industries, such as fuel storage, chemistry and catalysis. The application of MOFs in drug encapsulation and delivery systems is promising, especially in anti-inflammatory and anti-inflammatory drugs. Future research will focus on expanding the range of drug compounds that can be encapsulated in MOFs, enabling efficient delivery and controlled release of various therapeutic agents. Effective drug delivery can be greatly improved due to the potential for flexibility in drug delivery. In addition, MOFs have been widely reported in the field of biosensing. The material's distinct composition, tunability and adaptability make it perfect for sensitive and effective biosensors. Technology and in-depth chemical research will be necessary to guarantee the safety of MOFs for human usage and to advance their commercialization. MOFs will transform biomedical research, diagnosis and therapy by carrying out more research and encouraging innovation, hence enhancing human consumption of health and healing. Even though a lot of work has gone into developing technologies that use MOFs, the various research methodologies still make it challenging to understand how MOFs can be used for guiding. As a result, following the rules is essential to maintaining tight control over environmental safety, food quality and diagnostics.

Computer modeling-guided design could help create MOFs with better properties. One of the main challenges of MOF-based energy storage is maintaining stability under heavy use. Research should be conducted to create more stable MOFs, especially in humidity, high pressure and chemical reactions. Encapsulation and protection layer technology could also be explored. Advances in battery and supercapacitor technology should benefit from MOF-based electrode materials. To improve electrical performance and electrical cost, researchers need to examine the integration of MOFs with other electronic materials or create composite structures. This may include creating MOF-based electronic devices with different porosities and compositions. Advanced *in situ* characterization methods can provide insight into the electrochemical behavior of MOF-based electronic devices ion dynamics. This information can be used to guide the development of new materials and technologies. Despite the good properties of MOFs, their large-scale synthesis remains a challenge. Research is needed to improve the fabrication process, increase efficiency and build production capacity for MOFs to be used in energy storage applications. Collaboration between data scientists and engineers is important in this case.

## 8. Conclusions

MOFs are novel materials that can be applied to future energy storage systems points of view because they possess advantages like flexibility, high surface area and adjustable pore size. Such features enable enhanced density of the mobile ions, increased storage capacity and flexibility for changes in volume during charge–discharge cycles making them very suitable for use in batteries, SCs and other storage applications. The MOFs can easily be tuned to the prevailing environmental conditions and can be engineered at the molecular level consequently presents other merits in enhancing performance depending on the exact energy storage necessity. MOFs should be focused in the areas of their structure design, synthesis and investigation. During research, MOFs have the potential to be at the forefront in the designs and synthesis of efficient, robust and high-performance energy storage devices in sustainable energy technology. The development of these materials for uses, including industrial processes, energy storage, pharmaceutical delivery, electrical devices and environmental cleanup, will ultimately be fueled by advances in understanding of industries. Utilizing MOFs successfully in wearable energy storage devices will enable the production of safer and more ecologically friendly products in the future.

## Author contributions

Tholkappiyan Ramachandran: writing – review and editing, writing – original draft, methodology, investigation, S. V. Prabhakar Vattikuti: funding acquisition, writing – review and editing, Drafting, Yedluri Anil Kumar: writing – review and editing, writing – original draft, investigation, formal analysis, Sunkara Srinivasa Rao, Laxman Singh: formal analysis, Kwun Nam Hui, Duc Anh Dinh: supervision, formal analysis.

## Conflicts of interest

There are no conflicts to declare.

## Data Availability

No primary research results, software or code have been included and no new data were generated or analysed as part of this review.
